# Emerging Potential of Exosomal Non-coding RNA in Parkinson’s Disease: A Review

**DOI:** 10.3389/fnagi.2022.819836

**Published:** 2022-03-10

**Authors:** Peng Zhang, Madiha Rasheed, Junhan Liang, Chaolei Wang, Lin Feng, Zixuan Chen

**Affiliations:** ^1^School of Mechanical Engineering and Automation, Beihang University, Beijing, China; ^2^School of Life Sciences, Beijing Institute of Technology, Beijing, China; ^3^Beijing Advanced Innovation Center for Biomedical Engineering, Beihang University, Beijing, China

**Keywords:** Parkinson’s disease, exosome, non-coding RNAs, pathogenesis, diagnosis, treatment

## Abstract

Exosomes are extracellular vesicles that are released by cells and circulate freely in body fluids. Under physiological and pathological conditions, they serve as cargo for various biological substances such as nucleotides (DNA, RNA, ncRNA), lipids, and proteins. Recently, exosomes have been revealed to have an important role in the pathophysiology of several neurodegenerative illnesses, including Parkinson’s disease (PD). When secreted from damaged neurons, these exosomes are enriched in non-coding RNAs (e.g., miRNAs, lncRNAs, and circRNAs) and display wide distribution characteristics in the brain and periphery, bridging the gap between normal neuronal function and disease pathology. However, the current status of ncRNAs carried in exosomes regulating neuroprotection and PD pathogenesis lacks a systematic summary. Therefore, this review discussed the significance of ncRNAs exosomes in maintaining the normal neuron function and their pathogenic role in PD progression. Additionally, we have emphasized the importance of ncRNAs exosomes as potential non-invasive diagnostic and screening agents for the early detection of PD. Moreover, bioengineered exosomes are proposed to be used as drug carriers for targeted delivery of RNA interference molecules across the blood-brain barrier without immune system interference. Overall, this review highlighted the diverse characteristics of ncRNA exosomes, which may aid researchers in characterizing future exosome-based biomarkers for early PD diagnosis and tailored PD medicines.

## Introduction

Parkinson’s disease (PD) is the second most common neurodegenerative disease after Alzheimer’s disease (AD) (Reddy et al., 2020). PD has become an alarming concern due to its rapid increase and is estimated to rise about 10 million by 2030 globally (Vijiaratnam et al., 2021). The disease often occurs in middle-aged and older people, displaying clinical manifestations including motor symptoms: bradykinesia, muscle rigidity, resting tremor, and non-motor symptoms; loss of smell, sleep disturbance, and constipation (Jurado-Coronel et al., 2018). It has jeopardized the life of PD patients and reported extensive pressure on paramedical staff, which altogether causes a huge social burden (Bloem et al., 2021). The main pathological causes of PD include the degeneration and death of dopaminergic neurons in the substantia nigra pars compacta (SNpc) of the midbrain, resulting in the restricted delivery of dopamine (DA) to the striatum (Zhou et al., 2021). Thus, making a complex etiology PD, whose pathogenic mechanism is still not clear. It is speculated that it may involve abnormal regulation of α-synuclein (α-syn), mitochondrial dysfunction, oxidative stress, immuno-inflammatory mechanisms, excessive accumulation of neuromelanin, gastrointestinal-related dysfunction, and many other interactions (Yu et al., 2020).

**FIGURE 1:**
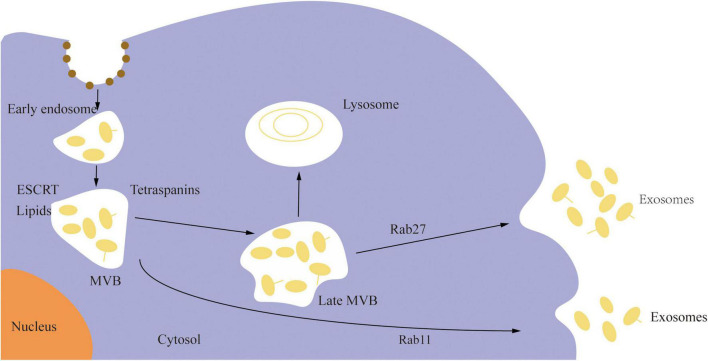
The biogenesis of exosomes.

Extracellular vesicles (EVs) are membrane cargo carriers secreted by cells and circulate in all body fluids. Under physiological and pathological situations, they exchange numerous components across cells, including nucleotides (non-coding RNAs, mRNA, DNA), proteins, and lipids, functioning as signal carriers (Thery et al., 2018; Pashova et al., 2020). These vesicles are categorized into three types based on their source or size: apoptotic bodies, microvesicles, and exosomes (Ciregia et al., 2017). Exosomes are lipid bilayer EVs, having a diameter of 40–160 nm (average 100 nm) and a 1.13–1.19 g/mL density, usually enriched with non-coding RNAs (ncRNA) (Kourembanas, 2015). They are the key mediator of several biological processes and are derived from the endosomal pathways. The plasma membrane invades to form an early sorted endosome, and the surface membrane of the late sorted endosome invades twice to form intraluminal vesicles after being changed into a multivesicular body. As shown in Figure 1, exosomes are formed when the multivesicular body unites with the cell membrane and is discharged into the extracellular space (Kalluri and LeBleu, 2020). Exosomes are widely distributed in cerebrospinal fluid and peripheral body fluids, where they play a potential role in several physiological functions such as signal transduction between cells, the transfer of pathogens, and the removal of cell debris. They are essential for the maintenance of neverous system development and function. Studies have shown that exosomes released by neurons can regulate synapses, neurodevelopment, neuroimmunity, and neuromuscular nodes; at the same time, exosomes released by oligodendrocytes, schwann cells, astrocytes, microglia, mesenchymal stromal stem cells, epithelial cells, and embryonic cerebrospinal fluid sources can maintain the normal function of neurons and neural stem cells (Janas et al., 2016).

Although the pathological aspects of PD require extensive research, recent studies have demonstrated that one of its pathological hallmarks is the accumulation of certain proteins in the brains of PD patients, specifically the buildup of DA neurotoxic alpha-synuclein (α-syn) (Schaeffer et al., 2020). Previously, it was thought that α-syn only had a harmful effect in cells; however, El-Agnaf et al. discovered that exosomes harboring α-syn in plasma and cerebrospinal fluid could disseminate in the brain (El-Agnaf et al., 2003). Converging studies have shown that α-syn can rely on exosomes to move from one neuron to another and form aggregates, causing neuronal apoptosis. These findings imply that exosomes containing α-syn may aid PD pathogenesis (Chistiakov and Chistiakov, 2017). Furthermore, the role of microglial exosomes in mediating the spread of α-syn cells, as well as the synergy between the inflammatory response and exosome-mediated α-syn diffusion, suggests that α-syn-containing microglial exosomes can produce classic PD lesions (Guo M. et al., 2020). Simultaneously, the α-syn deposition in glial cells can induce inflammation and spread to other glial cells and neurons. Neuroinflammation causes neuronal loss and worsens the development of PD (Chistiakov and Chistiakov, 2017).

Since the discovery of non-coding RNAs in exosomes, especially miRNAs (average length of 22 nucleotides) (Valadi et al., 2007), other non-coding RNAs such as long non-coding RNA (lncRNA, >200 nucleotides in length) and circular RNA (circRNA, covalently closed-loop structure, lacking a poly-A tail or 5 + to 3 + polarity) have also been recruited from exosomes of PD patients, speculating a prominent role of ncRNAs exosome in the pathogenesis of PD (Yin et al., 2019; Kuo et al., 2021; Qiu et al., 2021). These reported ncRNAs displayed important functions in cell development and metabolism, involving immune response, neurodevelopment, DNA repair, apoptosis, oxidative stress, and cancer (Allegra et al., 2012; Su et al., 2018). Mounting studies have shown that ncRNAs are essential for maintaining the normal function and integrity of the nervous system, and the dysregulated expression of these ncRNA results in PD pathogenesis (Juzwik et al., 2018, 2019).

**FIGURE 2:**
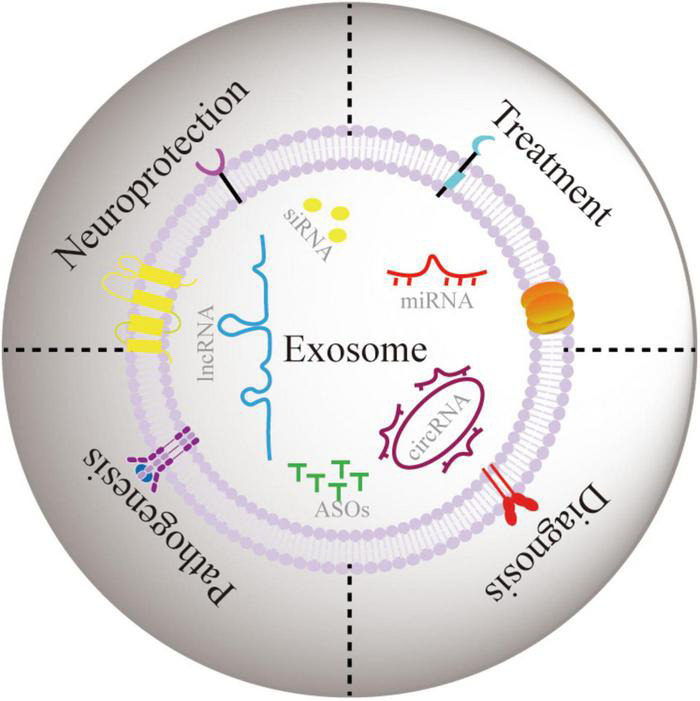
The role of exosomal ncRNA in neuroprotection, and pathogenesis, diagnosis, and treatment of PD.

So far, owing to the diversified distribution and dysregulation of ncRNAs in PD development, the potential role ncRNAs exosomes in PD progression is still not systematically reviewed. Henceforth, we performed categoric literature research to describe the association between exosomes and PD progression and the potential role of ncRNA exosome in neuronal function. As shown in Figure 2, we conducted a thorough investigation into the role of exosomal ncRNAs in the development, diagnosis and treatment of PD. Furthermore, we highlighted the critical role of exosomal ncRNA in neuroprotection to identify the shared mode of exosomal ncRNA regulation in PD.

## Nerroprotection of Exosomal Non-Coding RNA

### Synaptic Function

ncRNAs play an indispensable role in regulating normal brain development and function and the genesis of neurodevelopmental diseases (Rennert and Ziats, 2017; Zhang et al., 2019). Using an electron microscope, Lachenal et al. observed the release of exosomes from somato-dendritic compartments. The endosomal origin of exosomes shows that the C-terminal domain of tetanus toxin is specifically endocytosed by neurons and accumulates in multivesicular bodies, and is released in the extracellular medium together with exosomes. The release of such exosomes is regulated by calcium influx and glutathione synaptic activity, which indicates the role of exosomes in synaptic function (Lachenal et al., 2011). In another investigation, Morel et al. demonstrated that cortical neurons secrete miR-124a-containing exosomes, which can be directly internalized into astrocytes and thus rise astrocyte miR -124a and GLT1 protein levels (Morel et al., 2013). In another investigation, Morel et al. demonstrated that cortical neurons secrete miR-124a-containing exosomes, which can be directly internalized into astrocytes and thus rise astrocyte miR -124a and GLT1 protein levels (Men et al., 2019). Recently, Gao et al. reported that using endothelium cell exosomes to treat mice with induced ischemia/reperfusion injury, the intervention group’s foot failure rate was much lower than the control group’s (Gao et al., 2020). Additional analysis revealed that synaptic transmission, synaptic plasticity regulation, and synaptic vesicle cycle regulation were significantly more abundant in the intervention group than in the control group. For instance, up-regulation of synapsin-I expression in the motor cortex increases dendritic length. Moreover, miR-126-3p is found to protect PC12 cells from apoptosis and enhance neurite outgrowth, indicating that exosomes play an active role in changing the brain’s plasticity. Studies have shown that long-non-coding RNA (lncRNA) and circular RNA (circRNA) are equally important for neuron function (Ang et al., 2020). Bernard et al. characterized a lncRNA metastasis-associated lung adenocarcinoma transcript 1 (Malat1) abundantly expressed in neurons (Bernard et al., 2010). Knockdown of Malat1 in hippocampal neurons reduced synapse density, and its overexpression resulted in a cell-autonomous increase in synapse density. Chen et al. reported whole-transcriptome sequencing technology to systematically study the competing endogenous RNA (ceRNA) network in the rat hippocampus after PM2.5 exposure and identified 100 circRNA, 67 lncRNA, 28 miRNA, and 539 mRNA (Chen X. W. et al., 2021). Gene Ontology (GO) and Kyoto encyclopedia of genes and genomes (KEGG) analysis showed that these molecules are involved in synapses, neural projection, and neural development, particularly involved in signal pathways such as synaptic vesicle circulation. Recently, Fang et al. isolated circulating exosomes from blood samples of autistic infants and women with different physiopathological pregnancies (Fang et al., 2021). They observed that various lncRNAs-mRNAs related to synaptic vesicles (SV)-associated transcripts (SVATs) in autistic infants were differentially expressed from the first trimester of pregnancy to delivery. Thus speculates that pathological pregnancy issues may alter the GEP of SVAT and hence disrupt the intrauterine development of neural networks, thereby influencing fetal brain development (Fang et al., 2021). Taken together, ncRNAs encapsulated in exosomes play integral role in maintaining normal synaptic functions and can be used for to alter neuronal pathogenicities.

### Neuromuscular Junction

Neuromuscular junction (NMJ) is highly specialized synapse that formed between terminal end of motor nerve and a muscle fibers (cardiac/smooth, skeletal). They convert electrical impulses formed by motor neuron into electric signal in the muscle fibers. NMJ are usually protected by Schwann cells (SCs) and also represents the site of two-way chemical interaction between nerves and muscles. Interestingly, signals from muscles play an integral role in synapse formation, stability, maintenance, and function (Stanga et al., 2021). MotomiRs are defined as miRNAs essential for the development, maintenance, regeneration and survival of motor neurons (Hawley et al., 2017). From past few years, motor neuron-specific miR-218 has gained attention due to its role in mouse development (Reichenstein et al., 2019). Amin et al. named a coordinated gene set TARGET218, which contains 333 types of motor neuron mRNAs regulated by miR-218 (Amin et al., 2015). In the follow-up studies, they generated a series of mice that expressed different levels of miR-218, such as a motor neuron-selective gene regulator was associated with motor neuron disease. A non-linearly responsive regulon exhibits a steep dose-dependent threshold of miR-218 inhibition, and when crossed, it lead to severe motor neuron synaptic failure and death (Amin et al., 2021). In the substantia nigra (SN) of PD rats, lower expression of miR-218-5p resulted in the overexpression of LIM and SH3 protein 1 (LASP1). Up-regulation of miR-218-5p or inhibition of LASP1 ameliorated the pathological damage of DA neurons and increased the number of tyrosine hydroxylase and deacetylvindoline acetyltransferase positive cells in the SN of PD rats. Additionally, elevated levels of miR-218-5p or depressed LASP1 inhibited the apoptosis and oxidative stress of DA neurons in the brain SN of PD rats. Furthermore, overexpression of miR-218-5p inhibited the expression of LASP1 in the brain SN of PD rats, speculating LASP1 as a direct target of miR-218-5p (Ma et al., 2021). Another investigation on amyotrophic lateral sclerosis (ALS) model rats demonstrated that miRNA of dying neuron species can directly change the glial phenotype, leading to astrocyte dysfunctioning through vesicles. Research by Hoye et al. showed that motor neuron-derived miR-218 can be taken up by astrocytes and is sufficient to down-regulate an important glutamate transporter in astrocytes [excitatory amino acid transporter 2 (EAAT2)] (Hoye et al., 2018). However, the effect of miR-218 on astrocytes is not limited to EAAT2 due to multiple binding sites of miR-218 on diferent mRNAs, which altogether downregulated the translation of mRNAa in ALS astrocytes. Furthermore, neuronal miR-124-3p can be transferred to astrocytes through secreted neuronal exosomes to up-regulate the glutamate transporter GLT1 expression in astrocytes (Morel et al., 2013). A recent study on miR-124 in ALS rats model has reported the intercellular localization of miR-124-3p in the SOD1G93A, as well as a strong positive correlation between the level of exosomal miR-124-3p in the cerebrospinal fluid and the disease severity in (male) human ALS patients (Yelick et al., 2020).

### Neurodevelopment

Neural and non-neural EVs play a vital role in physiological and pathological neurodevelopment (Gomes et al., 2020). A study performed by Prieto-Fernandez on infant’s cerebrospinal fluid has identified 281 dysregulated miRNAs, out of which about 12 miRNAs were found in exosomes, speculating an important correlation between ncRNA in exosomes and neurodevelopment (Prieto-Fernandez et al., 2019). The neuronal innate immune response, which may affect neurodevelopment and neurodegeneration by regulating neuronal morphology, was linked with neuronal exosomes. Perceptible levels of Let7c and miR-21 was observed in neuronal exosomes and the developing brain. Both miRNAs interact with Toll-like receptor 7 (TLR7) to limit the dendrites growth but not neuronal growth (Liu et al., 2015). Goetzl et al. (2019) used the isolation of fetal central nervous system (CNS)-derived extracellular vesicles (FCE) from maternal plasma as a new method for non-invasive research on fetal neurodevelopment in early pregnancy. The results showed that the expression levels of synaptophysin, synaptophysin, synaptopodrin, and neurogranin in fetal central nervous system-derived extracellular vesicles (FCEs) of pregnant women exposed to heavy ethanol was significantly decreased (*P* < 0.001 for all) and the inhibition level of miR-9 in fetal FCE is tenfold high (90%). Furthermore, Shi et al. reported several lncRNAs in exosomes involved in regulating neural stem cell differentiation (Brn1b, RMST and TUNA), neuron proliferation (Pnky and Pou3f2), GABAergic neurons differentiation (EVF2), oligodendrocyte lineage (Sox8OT and Nkx2.2AS) (Shi et al., 2017).

### Neuroimmune Function

The inflammatory response is the most influencing biological process which regulates neurodevelopment and neurodegeneration. Mounting evidence has shown that EVs acts as a key mediator in the central nervous system communication that actively responds to nervoual damage, mediating inflammation and inflammation-related neuroprotection (Delpech et al., 2019). Lewy body formation (LBs) is the underlying neuropathological feature of PD (Shin and Chung, 2020), mainly composed of αlpha-synuclein. Under normal conditions, α-synuclein exists in a soluble form. However, under fatal pathological conditions, α-syn forms insoluble aggregates, leading to the gradual degeneration of neurons, which in turn promotes the development of PD. Misfolded proteins in the brains of people suffering from neurodegenerative disorders act as prions, spreading from one area to another (Jucker and Walker, 2013). Endosomes containing α-syn are transformed into mature late endosomes with the help of VPS4 and Small Ubiquitin-like Modifier proteins and fused with the plasma membrane to be secreted as exosomes; or, they can be classified as circulating endosomes and rely on Rab11a is exomitted as a secretory granule (Yu et al., 2020). Studies have shown that α-syn occurs in two states: linked to exosomes and free. Exosomal-associated α-syn, on the other hand, is more likely to be taken up by recipient cells and can cause more damage (Danzer et al., 2012). After 14 years of fetal SN transplantation into the PD’s patient striatum, researchers found that the transplanted fetal SN neurons contained Lewy body-like inclusions, stained positive for α-syn and ubiquitin, and immunostaining for DA transporters was reduced. It demonstrated that misfolded α-syn can spread from damaged cells to healthy transplanted cells (Kordower et al., 2008). Further, *in vitro* experiments have shown that exosomes released from SH-SY5Y cells overexpressing α-syn can effectively transmit α-syn to normal SH-SY5Y cells (Alvarez-Erviti et al., 2011).

Furthermore, neuron-derived exosomes can communicate with one another in nerve cells and glial cells to spread α-syn. The inflammation caused by α-syn accumulation in glial cells is transferred to other neurons and glial cells to exacerbate PD pathogenesis (Chistiakov and Chistiakov, 2017; Zheng and Zhang, 2021). Recently, Meng et al. reported that methamphetamine neurotoxic α-syn moves from neuronal cells to astrocytes via exosomes and triggers neuroinflammation (Meng et al., 2020). Guo et al. treated microglia with human α-syn prepared fibrils, and the released exosomes were able to promote protein aggregation in the recipient neurons. However, when coupled with microglia pro-inflammatory cytokines, it enhanced protein aggregation in neurons. Thus combined with animal studies, it is further confirmed that microglial exosomes contribute to the pathogenic progression of α-syn in PD (Guo M. et al., 2020).

**FIGURE 3:**
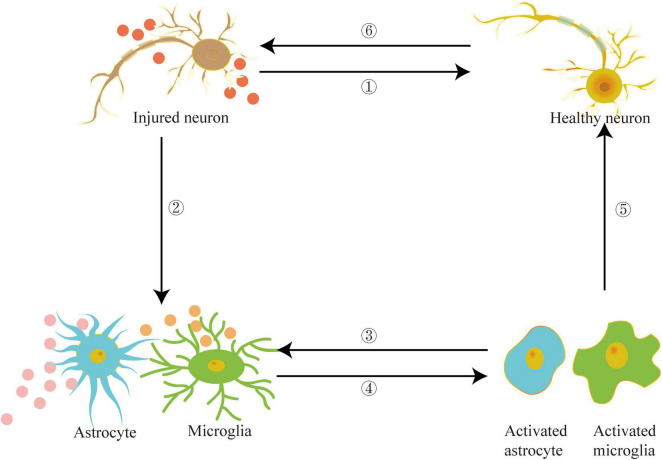
Exosomes can act as mediators between damaged neurons and healthy neurons, astrocytes, microglia and other target cells, promote inflammation and aggravate the development of PD.

Recently, the peripheral system, particularly blood abundant in exosomes, and its role in the etiology of PD have garnered attention. Han et al. found that the exosomes in the serum of PD patients contain increased levels of α-syn and inflammatory factors, which lead to the accumulation of α-syn, ubiquitin, and P62 in the recipient cells. Furthermore, PD patients’ serum exosomes had caused protein aggregation in the treated mice and stimulated DA neuronal degeneration, activated microglia, and caused apomorphine-induced rotation and movement defects (Han et al., 2019). Exosomes in the serum of PD patients had also stimulated microglia, causing them to lose autophagy. Thus higher accumulation of α-syn in microglia and accelerated secretion of α-syn into the extracellular space aided the transfer of α-syn to neurons (Xia et al., 2019). It seems that exosomes carrying α-syn to promote the etiology of PD have been widely recognized. However, a recent study found that after injecting α-syn-containing exosomes isolated from the brains of transgenic A53T mice into the striatum of wild-type mice, no endogenous α-syn accumulation was observed (Karampetsou et al., 2020). Researchers found no LBs like pathology in mice injected with recombinant fibril α-syn loaded with brain exosomes even after administration for seven months. As a result, it is hypothesized that even after long-term incubation, such exosomes are insufficient to damage neurons and drive the pathogenesis of PD. Figure 3 depicts that exosomes can act as a medium for communication between neurons and glial cells. Exosomes carrying PD pathogenic factors are released from damaged neurons and transferred to the targeted cells, such as healthy neurons, astrocytes, and microglia. The exosomes secreted by damaged glial cells further aggravate the inflammatory response, exacerbating nerve damage and promoting PD pathogenesis.

## The Diagnostic Implications of Exosomal Non-Coding RNA on Parkinson’s Disease

As PD symptoms primarily manifest in the late stages, identifying early biomarkers of PD has become crucial. However, due to a lack of suitable disease biomarkers and evident clinical symptoms in the early stages of PD, early clinical diagnosis has become more difficult. Exosomes, reported as an important mediator role in the pathogenesis of PD, are widely present in urine, plasma, bronchoalveolar lavage fluid, breast milk, and cerebrospinal fluid (Li et al., 2018). Previous studies have revealed that SNCA Hypomethylation, DJ-1, α-syn, Leucine-rich repeat kinase 2 (LRRK2), and acetylcholinesterase (AChE) have the potential to be early biomarkers of PD in exosomes (Fraser et al., 2016; Zhao et al., 2019, 2020; Shim et al., 2021). On the other hand, exosomes also contain ncRNAs that are highly connected to PD, implying that ncRNAs in exosomes have limitless potential in diagnosis (Fiedler et al., 2018). The potential biomarkers of ncRNA in the exosome of PD are summarized in Table 1 (ROC: Receiver Operating Characteristic Curve; AUC: Area Under the Curve).

**TABLE 1:** Potential biomarkers of ncRNA in exosome of PD.

Sample size	Exosome	ncRNA ID	Fings	ROC analysis	References
A = 40 B = 40	CSF	Let-7f-5p and miR-125a-5p	Increased in PD patients	AUC = 0.82 C = 90% D = 80%	Dos Santos et al., 2018
		miR-27a-3p,miR-423-5p and miR-151a-3p	Decreased in PD patients		
A = 40 B = 40	CSF	miR-10b-5p and miR-151a-3p	Increased in PD patients	AUC = 0.96 C = 97% D = 90%	Dos Santos et al., 2018
		miR-22-3p	Decreased in PD patients		
A = 27 B = 47	CSF	miR-153,miR-409-3p and miR-10a-5p	Increased in PD patients	AUC = 0.705-0.970 C = 93-95% D = 93-95%	Gui et al., 2015
		miR-1 and miR-19b-3p	Decreased in PD patients		
A = 109 B = 40	Serum	miR-19b	Decreased in PD patients	AUC = 0.753,C = 68.8% D = 77.5%	Cao et al., 2017
		miR-24	Increased in PD patients	AUC = 0.908,C = 81.7% D = 85.0%	
		miR-195	Increased in PD patients	AUC = 0.697,C = 82.6% D = 55.0%	
A = 25 B = 25	Serum	miR-29c	Increased significantly in PD patients	AUC = 0.689 C = 54.9% D = 80.0%	Ozdilek and Demircan, 2020
A = 52 B = 48	Plasma	miR-331-5p	Increased in PD patients	AUC = 0.849	Yao et al., 2018
		miR-505	Decreased in PD patients	AUC = 0.898	
A = 7 B = 34	Plasma	Let-7e-5p	Increased in PD patients	N/A	Nie et al., 2020
A = 32 B = 13	Plasma	lnc-MKRN2-42:1	Increased in PD patients	N/A	Wang et al., 2020
A = 51 B = 20	Plasma	POU3F3	Increased significantly in PD patients	AUC = 0.736 C = 68% D = 72%	Zou et al., 2020

*A for PD patients, B for Healthy control, C for Sensitivity, D for Specificity, CSF for Cerebrospinal Fluid, N/A for missing data.*

### The Separation and Extraction of Exosomes

For the diagnosis and treatment of PD, a prominent problem is the separation and extraction of exosomes from body fluids. At present, strategies such as differential centrifugation (DC), size exclusion, chromatography, ultrafiltration, precision, immunoaffinity capture and microfluidics have been developed (Bruce et al., 2019; Jeppesen et al., 2019). Among them, DC is the most commonly used method, which is suitable for large volume samples and has been used to purify exosomes from cell culture medium, plasma and urine. However, the high cost of the instrument, the long-time consumption of several hours, the low yield and high protein pollutants caused by the destruction of the integrity of the exosomes limit the popularization and application of DC. In order to improve the shortcomings of DC and improve the yield and purity of exosomes, density gradient ultrasound (DGC) strategies such as isopycnic UC and moving zone UC came into being (Gupta et al., 2018; Ding et al., 2021). Ultrafiltration based on nano membrane has the characteristics of simple operation, but there are high abundance of microbubbles, apoptotic bodies and protein contamination. In order to improve the protein concentration, another size exclusion chromatography strategy based on filtering came into being. This method has the advantages of simple operation, high yield and good reproducibility. However, the limitations of this method lie in the sample volume and yield (Ayala-Mar et al., 2019). The main application principle of precipitation is realized by polyethylene glycol and other water removing polymers. The purpose of separating precipitates rich in exosomes can be achieved simply by low-speed centrifugation or filtration (Popovic and de Marco, 2018). However, the large amount of coprecipitation of non-exosome pollutants (such as protein and polymer materials) is the main problem faced by this method. In addition, immunoaffinity capture has the advantage of separating exosomes with specific surface proteins. However, many other substances with this biomarker are also captured in the process of immune affinity, resulting in low purity of exosomes. Jalaludin et al. summarized the currently commercially available size exclusion chromatography kits including qEV (iZON), PURE-EVs (Hansa Biomed), and ExoLutE exosome isolation kit (Rosetta Exosome), precipitation kits including Exoquick (System Biosciences) and Total Exosome Isolation Kit (Thermo Fisher Scientific), and immunoaffinity capture kits including ExoQuant (Biovision), ExoCap Streptavidin Kit (MBL International), and Exosome Isolation Kit Pan (Miltenyi Biotec) (Jalaludin et al., 2021). In order to effectively separate exosomes and minimize time, equipment and cost, microfluidics strategies based on electrophoresis, dielectrophoresis, acoustics, magnetism and immune affinity came into being (Ding et al., 2021). However, the standardization, purity, retention of good biological function and related clinical validation of extracted exosomes remain to be studied. In addition, the integration strategy of high-throughput, fast, cost-effective and automation technology cannot be ignored. In short, each strategy for separating and purifying exosomes has its own advantages and disadvantages, which cannot be simply summarized as better.

### The Diagnostic Implications of miRNAs

Circulating miRNAs are strongly associated to the pathophysiological process of PD and can be collected simply using non- or minimally invasive techniques, making it a promising biomarker candidate for PD (Roser et al., 2018). Chen et al. examined plasma samples from healthy controls and PD patients using miRNA screening and analysis and discovered that miR-27a was up-regulated and let-7a, let-7f, miR-142-3p, and miR-222 were down-regulated, with AUC values all more than 0.8 (Chen et al., 2018). Khoo et al. investigated a series of PD prognostic biomarkers in the plasma circulating miRNAs of 32 PD/32 healthy controls, including k-TSP1 (miR-1826/miR-450b-3p), miR-626, and miR-505 (Khoo et al., 2012). After evaluating a new replication set of 42 PD/30 controls, it can achieve the highest predictive power of 91% sensitivity, 100% specificity, 100% positive predicted value, and 88% negative predicted value. Sulaiman et al. compared early-onset PD, plasma miRNA between late-onset PD and healthy controls (Sulaiman et al., 2020). The results showed that the multiples of miR-301a-3p, miR-100-5p, miR-140-5p, miR-486-3p, and miR-143-3p ranged from 11.2 to 32.0. In addition, the up-regulated miR-29b-3p, and the down-regulated miR-297, miR-4462, miR-1909-5p, and miR-346 may belong only to early-onset PD. Furthermore, miR-1297 and miR-4465, which regulate the GABAA gene region, were up-regulated in the plasma circulating miRNAs of 35 PD/35 healthy controls in PD patients (Cokmus et al., 2019). Another investigation on 151 PD patients, 21 multiple system atrophy (MSA) patients, and 138 healthy controls with circulating miRNAs reported an elevated miR-133b and miR-221-3p having 84.8% sensitivity 88.9% specificity (Chen Q. H. et al., 2021). However, a combination of miR-133b, miR-221-3p, and miR-4454 could be employed as a non-invasive biomarker for PD.

Although the utility of peripheral miRNAs in the early clinical diagnosis of neurodegenerative disorders has been called into question, because miRNAs in the peripheral circulation are subject to influence from other peripheral circulation components, the test findings are skewed. Exosome miRNAs are highly stable and resistant to degradation. Thus proposes miRNA exosomes to be a potential biomarker for early detection (Dong et al., 2020). Dos Santos analyzed the miRNA carried by exosomes in 40 early PD patients and 40 control samples CSF using a cross-sectional cohort that included small RNA sequencing, protein binding ligand assays, and machine learning (Dos Santos et al., 2018). The results showed that the expression levels of Let-7f-5p and miR-125a-5p had increased, while expression levels of miR-27a-3p, miR-423-5p and miR-151a-3p decreased. The analysis of the binding of miRNA profile with PD marker protein showed that the expression level of miR-22-3p decreased, while the expression level of miR-10b-5p and miR-151a-3p increased. Gui et al. found that the expression of miR-1 and miR-19b-3p in the CSF of PD patients was significantly reduced, whereas the expression of miR-153 and miR-409-3p, miR-10a-5p and let-7g-3p expression were significantly increased (Gui et al., 2015). However, the combined analysis of miR-153 and miR-409-3p can significantly improve discrimination performance.

In comparison to extracting other body fluids such as blood, obtaining cerebrospinal fluid is more complicated, with higher risks and costs (Marrugo-Ramirez et al., 2021). Cao et al. found the possibility of miR-19b, miR-24 and miR-195 as PD diagnostic markers through a study of 109 PD patients and 40 healthy controls (Cao et al., 2017). By comparing the expression levels of miR-19a, miR-19b, miR-29a, miR-29c, miR-181, miR-195 and miR-221 in the serum of 51 PD patients and 20 healthy controls, Ozdilek et al. found that the expression level of miR-29c in PD patients increased significantly (Ozdilek and Demircan, 2020). The level of miR-195 is only significantly positively correlated with age. The level of miR-29a was significantly negatively correlated with the Unified Parkinson’s Disease Rating Scale (UPDRS) total score. After comparing the plasma exosomes samples of 52 PD patients and 48 healthy controls, Yao et al. found that the expression of miR-331-5p was significantly increased in PD patients, while the expression of miR-505 was significantly decreased (Yao et al., 2018). Another study showed that miR-125a-5p, miR-1468-5p, miR-204-5p, let-7e-5p, miR-375, miR-369-5p, miR-423-5p, and miR-23a-3p have a substantial increase/decrease in the plasma of 34 normal control groups, 5 AD donors, and 7 PD donors, according to Nie et al. (2020). Among them, the expression of Let-7e-5p is elevated in PD patients. Moreover, the up-regulated/down-regulated miRNAs in PD samples are enriched in fatty acid biosynthesis pathways. Additionally, we have found that putative PD biomarkers in urine and saliva have received extensive attention from researchers (Ho et al., 2014; Fraser et al., 2016; Cressatti et al., 2020; Figura et al., 2021). However, there are few reports on the investigation of exosomal ncRNA.

### The Diagnostic Implications of lncRNA

Kraus investigated the levels of expression of 90 well-annotated lncRNAs in brain samples from PD patients and healthy controls (Kraus et al., 2017). The expression of H19 upstream conserved 1 and 2, lincRNA-p21, Malat1, SNHG1, and TncRNA was shown to be considerably altered in PD patients. It reflects the critical role of lncRNA in the etiology and diagnosis of PD. Recently, Cheng et al. examined blood TUG1 levels in 97 PD patients (50/47) and 84 healthy controls (Cheng et al., 2021). The study’s findings revealed that serum TUG1 can distinguish PD patients from healthy controls (AUC = 0.902, Sensitivity = 88.7%, Specificity = 89.3%). TUG1 down-regulation improves motor coordination in PD mice while inhibiting the production of inflammation-related proteins, according to animal research. The expression of rhabdomyosarcoma 2-associated transcript (RMST) in the serum of PD patients rose as well (AUC = 0.892) (Chen C. L. et al., 2021), and it was positively correlated with the expression of inflammatory factors. miR-150-5p is the target gene of RMST. Knockdown of RMST had reduced the apoptosis and inflammation of SH-SY5Y cells. Furthermore, Quan et al. found that the expression level of maternally expressed gene-3 (MEG3) in the plasma of PD patients was lowereed than that of the anti-control group, and revealed the possibility of lncRNA MEG3 as a new candidate biomarker for PD (Quan et al., 2020).

Although research on lncRNA as a biomarker for the diagnosis, treatment, and prognosis of PD patients has advanced rapidly, there are still few studies on exosomal lncRNA (Taghizadeh et al., 2021). A study on Peripheral blood exosomal lncRNA expression levels in 32 PD patients and 13 healthy volunteers from Beijing Tiantan Hospital found 15 upregulated and 24 downregulated exosomal lncRNAs (Wang et al., 2020). Among them, the expression of lnc-MKRN2-42:1 was positively correlated with the MDS-UPDRS III score in PD patients. The changes of L1CAM in plasma exosomes are closely related to the health of the central nervous system (Shi et al., 2014). Zou et al. compared the activities of POU3F3, α-syn, and GCase in neuronal-derived blood exosomes containing L1 cell adhesion molecules (L1CAM) in 93 PD patients and 85 healthy controls (Zou et al., 2020). POU3F3 is significantly positively correlated with α-syn, and negatively correlated with GCase activity. ROC curve shows that when OU3F3, L1CAM and GCase are analyzed jointly, the combined variable is more reliable than the single variable.

It is of practical significance to diagnose PD by exosomal ncRNA. However, we found that the exosomes extracted in most studies were through commercial kits. Among them, whether the existence of non-secrete pollutants will affect the accuracy of result analysis remains to be studied. In addition, the current research lacks unified standards for sample collection, database construction and analysis. All these have caused great differences in the results between different studies.

## The Pathogenic Effect of Exosomal Non-Coding RNA on Parkinson’s Disease

**FIGURE 4:**
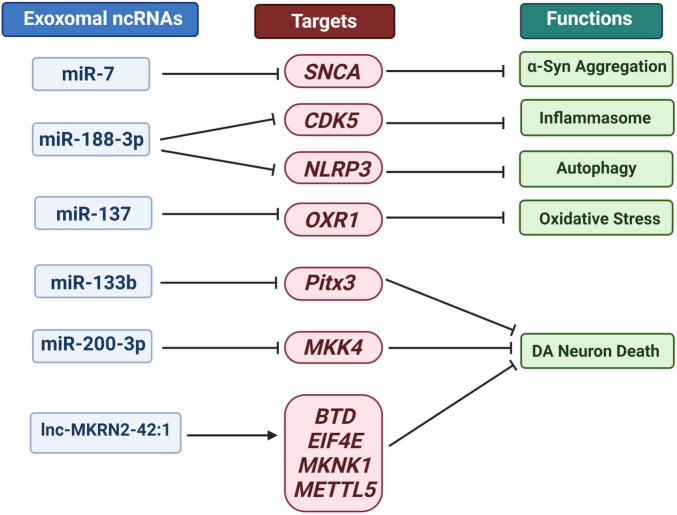
Overview of exosomal ncRNA in several PD related pathogenesis.

ncRNA can move between cells, causing alterations in the gene programs of target cells and thereby accelerating the progression of PD (Izco et al., 2021). The pathogenesis of PD involves oxidative stress, mitochondrial dysfunction, inflammation and α-syn aggregation. At the same time, a destructive cycle is established, which eventually leads to the degeneration of DA neurons (Sun et al., 2019). Harischandra expressed wild-type human α-syn in the MN9DDA cell model of PD to investigate the influence of manganese exosomes content and release (Harischandra et al., 2018). Exosomes were released into the extracellular media after 24 h of exposure, and the expression of Rab27a protein, which regulates the exosomes release, increased. Exosomes extracted from manganese-exposed cells contained more miRNAs according to RNA-Seq analysis. About 12 miRNAs including: miR-210-5p, miR-505-5p, miR-128-1-5p, miR-325-5p, miR-16-5p, miR-1306-5p, miR-669b-5p, miR-125b-5p, miR-450b-3p, miR-24-2-5p, miR6516-3p and miR-129 increased significantly by 2.5 to 15 times. These miRNAs were found closely associated with protein aggregation, autophagy, inflammation, and hypoxia. For example, as effective PD biomarkers, miR-16-2*, miR-26a2* and miR30a can distinguish between treated and untreated patients (Margis et al., 2011). miR-16 targets included α-syn and transferrin receptor 1 (Parsi et al., 2015). Additionally, Zhang et al. found that inhibiting heat shock protein 70 promoted the aggregation of α-syn in the human neuroblastoma cell line SH-SY5Y-Syn overexpressing α-syn. Simultaneously, miR-16-1 inhibits heat shock protein 70 and promotes α-syn aggregation in SH-SY5Y-Syn cells (Zhang and Cheng, 2014). In Figure 4, we summarize the role of exosomal ncRNA in PD related pathogenesis.

### SNCA Accumulation and α-Syn Aggregation

Some miRNAs can bind to the 3′- untranslated region (UTR) of *SNCA* gene to inhibit its transcription and α- syn’s translation. Studies have shown that the loss of miR-7 and miR-34b/c lead to changes in the brain of PD patients α-syn aggregation and dopaminergic neuron loss (Kabaria et al., 2015; McMillan et al., 2017). MSC derived exosomes modified by mimic-miR-7 can inhibit α- syn aggregates and inhibits NLRP3 inflammasome activation in SNPC and striatum, thereby improving neuroinflammatory response in PD (Yu et al., 2020).

### The Activation of Autophagy and Inflammasome

Autophagy lysosomal pathway can promote the repair and removal of α-syn abnormal aggregation of PD (Pan et al., 2008). In addition, the occurrence of inflammation can actively promote the occurrence and development of neurodegeneration, including PD (Lopez Gonzalez et al., 2016). Li et al. evaluated the levels of injury, autophagy and inflammatory bodies in MPTP induced PD mouse model and MPP + induced cell model (Li et al., 2021). They found that miR-188-3p-rich adipose-derived stem cell (ADSC)-derived exons can suppress autophagy and pyrolysis in PD mice and MN9D cells by targeting CDK5 and NLRP3 (Li et al., 2021). Cyclin dependent kinase 5 (CDK5) is a serine/threonine kinase. CDK5 mediated phosphorylation of EndoB1 and raf kinase inhibitor protein (RKIP) is crucial for autophagy induction and neuronal loss in PD model (Wong et al., 2011; Wen et al., 2014). In addition, as the main component of chronic inflammation, NOD like receptor protein 3 (NLRP3) inflammatory bodies play a crucial role in PD through caspase 1 activation, which is mainly induced by mitochondrial damage (Yan et al., 2020). The expression of CDK5 and NLRP3 is not independent, but has a close correlation. Up regulation of CDK5 can increase the expression of BRCA1-BRCA2 containing complex subunit 3 (BRCC3) in HEK293 cells, while inhibition of CDK5 reduces the up-regulated BRCC3 level in 1-methyl-4-phenylpyridinium (MPP +) induced PD cell model (Cheng et al., 2020). In addition, their experiments confirmed that CDK5 mediated BRCC3 expression may play a key role in neuronal inflammation by regulating NLRP3 inflammatory bodies in PD.

### Oxidative Stress

The antioxidant 1 (OXR1) gene identified in the screening of human cDNA library is a gene that can save the DNA oxidative repair defect of spontaneous *E. coli* mutants (Volkert et al., 2000). Studies have shown that OXR1 plays an increasing role in neurodegenerative diseases such as oxygen-induced retinopathy, diabetic retinopathy, PD, ischemia-induced neuronal damage, and ALS due to its outstanding ability to reduce oxidative stress and neurodegeneration (Volkert and Crowley, 2020). Finelli et al. found that the decreased expression of OXR1 was related to the degeneration of brain granulosa cells (CGC), the progressive and rapid deterioration of motor coordination, and the shortening of life span (Finelli et al., 2019). The effect of OXR1 on motor function also suggests its potential therapeutic potential in PD. Studies have shown that miR-137 targeted OXR1 and negatively regulated its expression, and inhibiting miR-137 or up-regulating OXR1 can promote the vitality of neurons in PD models and minimize apoptosis, accompanied by a drop in MDA content and ROS levels and an increase in SOD levels (Jiang et al., 2019). Exosomes derived from Mesenchymal Stem Cells/Somatic Cells (MSC) can transport miRNAs to neuronal cells and are critical in immunological modulation, neurite remodeling, neurogenesis, and axonal growth and CNS recovery (Vilaca-Faria et al., 2019; Nasirishargh et al., 2021). Jiang et al. injected serum-derived exosomes into PD mice, and the loss of function of miR-137 antagomir demonstrated that miR-137 down-regulation could minimize PD’s oxidative stress damage by up-regulating OXR1, reduce pole-climbing time, and increasing traction test score (Jiang et al., 2019). OXR1 has a wide range of inhibitory effects on oxidative stress. In addition to its potential for the treatment of neurodegenerative diseases, OXR1 also shows excellent effects in delaying lupus related renal failure (Li et al., 2014).

### Dopamine Neuron Death

It is worth noting that FTY720 and its derivatives approved by the Food and Drug Administration (FDA) may increase miR376b-3p, miR-128-3p, miR-146b-5p, miR-7a-5p, miR-9- 5p and miR-3p 30d-5p expression to reduce the loss of α-syn and DA neurons in PD and related diseases (Vargas-Medrano et al., 2019). miR-128, which is particularly prevalent in the hippocampus, is expressed at higher levels in neurons than glial cells (Qiu et al., 2014). Tan et al. showed that miR-128 expressed in adult neurons can affect motor behavior by suppressing the production of numerous ion channels and signal components of the extracellular signal-regulated kinase ERK2 network, which modulates neuron excitability. In mice, increased expression of miR-128 had impaired neuronal reactivity, inhibited motor activity, and alleviated PD and epilepsy symptoms (Tan et al., 2013). Zhou et al. investigated how miR-128 regulates DA neuron death and the expression of excitatory amino acid transporter 4 (EAAT4) via binding to AXIN1 (AXIN1). The findings reveal that miR-128 can minimize DA neuron apoptosis, boost EAAT4 expression, and suppress AXIN1 expression (Zhou et al., 2018). Other studies have demonstrated that down-regulation of Axin1 can activate the HIF1/miR-128-3p axis, which then activates the Wnt/-catenin pathway, preventing hippocampus neurodegeneration in PD mice wounded by 1-methyl-4-phenyl-1,2,4,5-tetrahydropyridine (MPTP) (Zhang et al., 2020). circRNA is one of the most abundant ncRNAs in the brain, with a highly stable circular structure, and plays a vital role in neurological illnesses (Ma et al., 2020). RNA sequencing of multiple brain tissues from dozens of PD patients and control donors revealed that the accumulation of circRNA in the SN of PD patients was not age-dependent, such that the expression of circSLC8A1 increased with the RNA target of miR-128 (Hanan et al., 2020). circSLC8A1 has 7 binding sites for miR-128 and can strongly bind to the miRNA effector protein Ago2, indicating that circSLC8A1 affects the function and/or activity of miR-128 (Hanan et al., 2020). Even though studies have proven that PD is intimately tied to exosomes and ncRNA. However, there is currently no direct evidence to support the role of exosomal circRNA in the etiology of PD.

miR-133b was discovered to be selectively expressed in midbrain DA neurons (DNs) but not in PD midbrain tissue. miR-133 can regulate the maturation and function of dopaminergic neurons in the midbrain, increases neuronal density and weakens neuronal apoptosis (Kim et al., 2007). In a negative feedback loop involving the pair-like homeodomain transcription factor (Pitx3), miR-133b was reported to influence the maturation and function of midbrain DNs (Kim et al., 2007). New studies have found that MSCs derived exosomes can transfer miRNA to neuronal cells, and the exosomes rich in miR-133b can promote the growth of neural protrusion and improve the function of neurons (Vilaca-Faria et al., 2019).

Shakespear et al. (2020) demonstrated that exosomes released from normal astrocytes can significantly attenuate MPP + induced cell death in SH-SY5Y cells and primary mesencephalic dopaminergic neuron cultures in PD cell model. Small-RNA sequencing, target analysis, and reporter assay determined that miR-200a-3p targeted MKK4 by binding to two independent sites on the 3′- UTR of *Map2k4*/MKK4 mRNA and inhibited the expression of MKK4 mRNA and protein. There have been numerous reports on the beneficial effects of lncRNA in PD or traumatic brain injury (TBI) (Patel et al., 2018; Lun et al., 2022), however, the exosomal lncRNA is still an area that needs to be investigated and expanded in the field of PD treatment. The mixed lineage kinase (MLK) family of MAPKKKS activates the neuronal death signaling pathway by phosphorylating and activating downstream MAPK kinases (MKKs), and then induces the phosphorylation and activation of c-Jun N-terminal kinases (JNKs), which are the basic mediators of neuronal death observed in various neurodegenerative diseases (Chen et al., 2012). Autopsy studies, as well as animal models of MPTP and 6-OHDA neurodegeneration, show that JNK plays an important role in the pathogenesis of the disease (Iqbal et al., 2015). In a study evaluating the effect of catalpol on MPTP treated mice to establish PD model, the results showed that catalpol affected MKK4/JNK/cJun signaling pathway, reduced the loss of dopamine neurons, increased TH expression and delayed MPTP induced oxidative stress (Li and Zhao, 2021).

The RNA-Seq data analysis on white blood cells from PD patients before and after Deep Brain Stimulation (DBS) treatment revealed that U1 spliceosomal lncRNA and RP11-462G22.1 changed dramatically (Soreq et al., 2014). Kraus et al. examined the expression levels of 90 well-annotated lncRNAs in 30 brain specimens from 20 PD patients and 10 healthy controls for the first time, and discovered that H19 upstream conserved 1 and 2 were significantly down-regulated in PD patients, while lincRNA -p21, Malat1, SNHG1, and TncRNA were significantly up-regulated (Kraus et al., 2017). Recently, Wang et al. analyzed the expression of lncRNAs in peripheral blood vesicles in 32 PD patients and 13 healthy controls, identifying 15 up-regulated and 24 down-regulated lncRNAs. Among these, lnc-MKRN2-42:1 is associated with a higher MDS-UPDRS III score in PD patients. Bioinformatics analysis showed that lnc-MKRN2-42:1 could trans regulate *BTD,EIF4E,MKNK1,METTL5* and other target genes, and engages in biological activities such as apoptosis, synaptic remodeling, long-term potential, immunity, and glutamate neurotransmitter metabolism (Wang et al., 2020). lnc-MKRN2-42:1 is a newly discovered intergenic antisense lncRNA. There are few reports on it, and its specific function remains to be studied. Overall, these investigations provide a foundation for tracking the progression of PD and identifying prospective treatment targets.

It is undeniable that a large number of studies have shown that exosomal ncRNA plays an important role in the pathogenesis of PD. However, other substances in exosomes, such as α-syn propagation is also important to PD pathogenesis. At present, there is no study to clarify in detail whether the role of exosomal ncRNA is decisive. At the same time, the correlation between the effects of different PD related substances in exosome on the process of pathology is also lack of relevant research and discussion.

## The Treatment Implications of Exosomal Non-Coding RNA on PD

Clinical therapies for PD patients have considerably improved in recent years. However, there is still a dearth of effective treatments to cure and prevent the progression of PD in order to improve PD patients’ quality of life. Researchers have uncovered a number of novel targets and developed a variety of treatment strategies based on a better knowledge of the mechanism of PD, including gene therapy, α-syn targeted therapy, and glutathione replacement therapy (Charvin et al., 2018). The blood-brain barrier (BBB) is a selective barrier between blood circulation and the brain, consistent with endothelial cells with tight junctions, astrocyte end-feet surrounding the endothelium, pericytes, and the extracellular matrix’s basement membrane (Willis et al., 2013). Unlike high permeability peripheral capillaries, brain capillary endothelial cells (BBB endothelial cells) express a variety of transmembrane transporters and contain the major facilitator superfamily domain containing 2a (MFSD2A), which strictly limits the transfer of various substances from the blood to the brain (Liang and Chen, 2020). Changes in the structure and function of the pathological BBB have almost no effect on AD, PD, Huntington’s disease, amyotrophic lateral sclerosis, multiple sclerosis, HIV-1 related dementia and chronic traumatic encephalopathy (Sweeney et al., 2018). On the other hand, BBB represents a major obstacle for delivering therapeutic drugs for the central nervous system diseases to reach the brain because it limits the passage of 98% of small molecules with molecular mass less than 800 Da (Guo Y. D. et al., 2020). Mounting evidence has shown that EVs can cross biological barriers using endogenous intracellular transport mechanisms and trigger responses when ingested by recipient cells (Elsharkasy et al., 2020). Banks et al. used multiple regression analysis to examine the ability of 10 exosomal populations from mice, humans, cancer cells, and non-cancerous cell lines to cross the BBB, and reported that exosomes can cross the BBB at different rates and mechanisms (Banks et al., 2020). Furthermore, exosomes as drug carriers have the advantages of low immunogenicity, resulting in low cytotoxicity, high blood stability, high efficiency due to effective drug protection, good targeting and easy fusion with recipient cells; similar in structure to recipient cells and easily absorbed by a variety of cells (Peng et al., 2020). Convincing studies have shown that exosomes in the blood exhibited natural brain targeting ability related to the transferrin-transferrin receptor interaction. These exosomes have special effects in the treatment of PD, since they can deliver DA to the brain and significantly increase the DA content in the brain (Qu et al., 2018). Additionally, exosomal carriers play a prominent role in reducing DA neuronal apoptosis and boosting DA neurons repair and regeneration (Harrell et al., 2021). There have been numerous studies on the therapy of PD, including exosomal loading protein and mRNA (Haney et al., 2015; Kojima et al., 2018). Here we only summarize exosomal ncRNA (Table 2).

**TABLE 2:** Exosome is used as a carrier of ncRNA for the treatment of PD.

Exosome source	ncRNA	Experimental objects	Mechanisms	Brain regions	References
Murine dendritc cells	siRNA	Syn-SHSY5Y,S129D -syn transgenic C57BL/6 mice	Reduced syn-related mRNA and protein levels	Midbrain, striatum and cortex	Cooper et al., 2014
Immature dendritic cell	siRNA	SH-SY5Y,MPTP C57BL/6 mice	Inhibit the expression and aggregation of α-syn protein	SN	Liu et al., 2020
Murine dendritic cells	shRNA	Syn-SHSY5Y, S129D α-syn transgenic C57BL/6 mice	Reduced α-nuclide polymerization and loss of dopamine neurons	Striatal; frontal, somatosensory, and somatomotor cortex; amygdala; midbrain; SNc	Izco et al., 2019
Marrow stem cells	ASO	SH-SY5Y,HEK293,Primary neurons,α-Syn A53T mouse	Inhibit the expression and aggregation of α-syn protein	Striatum, SN	Yang et al., 2021

*N/A for missing data.*

### The Treatment Implications of siRNA

RNA interference (RNAi) is an effective mechanism to suppress gene expression at the post-transcriptional level. The 22-nucleotide (nt) small interfering RNA (siRNA) can inhibit the expression of messenger RNA (mRNA) (Sheng et al., 2020). However, siRNA has the disadvantage of poor stability, making naked siRNA delivery unfeasible (Sharma et al., 2020). Exosomes have significant advantages as a carrier in terms of siRNA protection, guidance, and delivery. Exosomes derived from rabies virus glycoprotein (RVG) peptide modified murine dendritic cells can specifically bind to neuronal cells and acetylcholine receptors expressed by BBB. Cooper et al. loaded siRNA with PD therapeutic effects into exosomes (Cooper et al., 2014). siRNA1, 2, and 3 significantly reduced total α-syn mRNA (55, 65, and 80% decrease, respectively), and protein (64, 68, and 85% decrease, respectively) in cell models. siRNA3 loaded with the most effective RVG exosomes was injected intravenously into 5-month-old Tg13 mice. It was found that the relevant mRNA levels in midbrain, striatum, and cortex were significantly reduced (49, 56, and 50% decrease, respectively), and S129D α-Syn-HA protein levels were significantly reduced (32, 26, and 30% decrease, respectively). Liu et al. designed a nanomaterial named RVG peptide–modified exosome (EXO) curcumin/phenylboronic acid-poly (2-(dimethylamino)ethyl acrylate) nanoparticle/small interfering RNA targeting SNCA (REXO-C/ANP/S) (Liu et al., 2020). The material has an engineering core-shell hybrid structure that simultaneously loads hydrophilic genes (siRNA) and hydrophobic small molecule drugs (curcumin). This delivery system showed a significant clearing effect on the TH + neuron α-syn in the SN area. Additionally, T cell activation of PD mice was significantly decreased, and the expression of Fox p3 in CD4 positive (CD4+) T cells was significantly increased. TGF- and IL-10 related to anti-inflammatory effects increased significantly, and IL-22 and IL-17 related to pro-inflammatory effects decreased significantly.

### The Treatment Implications of shRNA

siRNA has a relatively short half-life *in vivo*, which causes transient knockdown of its target protein (Baroy et al., 2010). Short hairpin RNAs (shRNAs) have a longer action time and are more stable in expression (Yang et al., 2019). For chronic diseases with a long onset duration such as PD, the long action time of shRNA is much more advantageous. To accomplish brain targeting, Izco et al. loaded shRNA into exosomes derived from murine dendritic cells modified with a brain-targeting peptide (rabies virus glycoprotein [RVG] peptide) (Izco et al., 2019). Furthermore, injecting anti-α-syn shRNA-MC encapsulated by RVG-exosomes into PD mice by intravenous injection has reduced frontal cortex (decreased 58%), somatosensory cortex (decreased 65%), and somatomotor cortex (decreased 80%), the amygdala (decreased 37%) and SNc (decreased 37%) after 90 days of treatment. Similarly, the expression of α-syn protein was considerably reduced when comparing the ipsilateral midbrain (decreased 54% compared to controls). Furthermore, the loss of DA neurons in the SNc in the PD model was as high as 75%, and the expression level of the shRNA treatment group was restored the same as the control group.

### The Treatment Implications of Antisense Oligonucleotides

RNAi has a good effect on reducing the α-syn expression, aggregation, and inflammation in PD; however, there are problems such as potential DA neuron degeneration (Collier et al., 2016). In contrast, antisense oligonucleotides (ASOs)-based gene therapy is more mature and reliable in treating a wide range of neurodegenerative diseases (Osmanovic et al., 2020). ASO ranges in length from 18 to 30 base pairs (bp), and the expression of target mRNA is modified by altering splicing or attracting RNase H, resulting in target destruction (Scoles et al., 2019). Many ASO medications are now in development testing or have been licensed by the FDA to treat movement disorders (Scoles and Pulst, 2019). Yang et al. screened the most effective ASO based on the human SNCA sequence and developed a safe and efficient ASO delivery method using exosomes (Yang et al., 2021). *In vitro* investigations demonstrated the delivery system’s high cellular absorption and low toxicity, while also reducing fibril-induced α-syn aggregation. *In vivo* tests also revealed that the administration approach dramatically lowers α-syn expression and aggregation, improves DA neurodegeneration, and significantly improves motor function.

## Conclusion

In conclusion, ncRNAs in exosomes are required for appropriate neuronal activity. The disruption of ncRNA expression in exosomes is likely to play an important role in the etiology of PD. However, there has been a dearth of particular and conclusive studies on the functional meaning of ncRNA in exosomes in the pathogenesis and progression of PD and the specific involvement in the pathogenesis of PD. Therefore, determining the importance of ncRNA in exosomes in the etiology of PD is a critical scientific topic that must be resolved as soon as possible. Exploring this issue can help us understand the process of PD development and progression and may also aid in the development of novel ways for diagnosis and treatment.

At present, different methods are usually combined to obtain better results of exosome extraction. Therefore, it is difficult to define the quality of an exosome separation and purification strategy. In a word, one of the outstanding problems of exosome treatment vector is to find a strategy for mass production of exosomes with high purity and good homogeneity. For diagnosis, the strategy of exosome extraction should be simple, rapid and accurate, so as to facilitate nucleic acid detection, biomarker screening and protein detection.

Exosomes are widely present in urine, plasma, bronchoalveolar lavage fluid, breast milk, and cerebrospinal fluid during the life cycle, and they carry a variety of ncRNAs that are highly connected to PD. The exosomal ncRNA has been extensively explored as an early biomarker of PD, which is important for the early diagnosis of the disease. However, the small number of samples and the potential medical conditions of the test subjects limits the research. The lack of inherent RNA as a housekeeping gene causes discrepancies between different studies. Furthermore, the issues of sample collection, database development, and analysis standardization have not yet been fully resolved.

Exosomal ncRNA has minimally invasive and long-term efficacy in treating PD, and it can be disseminated to the brain repeatedly during the treatment process, making it a very promising tool for the prevention and treatment of neurodegenerative illnesses. Exosomes, on the other hand, have complicated components and unknown potential roles. They may contain apoptotic bodies and microvesicles in exosome samples obtained from culture media; thus the purity of exosomes is one of the main concerns of researchers. However, natural exosomes, on the other hand, are easily destroyed by the immune system. These issues have posed difficulties for its pre-clinical treatment engineering.

Overall, we anticipate that the importance of exosomal ncRNA in neuroprotection and the etiology of PD will become more apparent as research advances. Furthermore, the ongoing development of sample collection, database construction, and analysis standardization may provide new hope for the early detection of exosomal ncRNA in PD. Diagnostic and therapeutic strategies involving exosomes ncRNA will become dominant in the future due to the active participation of scientists in engineering exosomes modifications and large-scale production methods.

## Author Contributions

PZ and ZC designed and drafted the sections of the manuscript. PZ prepared the primary manuscript and tables. JL and CW prepared the figures. MR, LF, and ZC revised the English. All authors read and approved the final manuscript.

## Conflict of Interest

The authors declare that the research was conducted in the absence of any commercial or financial relationships that could be construed as a potential conflict of interest.

## Publisher’s Note

All claims expressed in this article are solely those of the authors and do not necessarily represent those of their affiliated organizations, or those of the publisher, the editors and the reviewers. Any product that may be evaluated in this article, or claim that may be made by its manufacturer, is not guaranteed or endorsed by the publisher.

## References

[B1] Allegra A. Alonci A. Campo S. Penna G. Petrungaro A. Gerace D. (2012). Circulating microRNAs: new biomarkers in diagnosis, prognosis and treatment of cancer (Review). *Int. J. Oncol.* 41 1897–1912. 10.3892/ijo.2012.1647 23026890

[B2] Alvarez-Erviti L. Seow Y. Schapira A. H. Gardiner C. Sargent I. L. Wood M. J. (2011). Lysosomal dysfunction increases exosome-mediated alpha-synuclein release and transmission. *Neurobiol. Dis.* 42 360–367. 10.1016/j.nbd.2011.01.029 21303699 PMC3107939

[B3] Amin N. D. Bai G. Klug J. R. Bonanomi D. Pankratz M. T. Gifford W. D. (2015). Loss of motoneuron-specific microRNA-218 causes systemic neuromuscular failure. *Science* 350 1525–1529. 10.1126/science.aad2509 26680198 PMC4913787

[B4] Amin N. D. Senturk G. Costaguta G. Driscoll S. O’Leary B. Bonanomi D. (2021). A hidden threshold in motor neuron gene networks revealed by modulation of miR-218 dose. *Neuron* 109 3252–3267.e6. 10.1016/j.neuron.2021.07.028 34450025 PMC8542606

[B5] Ang C. E. Trevino A. E. Chang H. Y. (2020). Diverse lncRNA mechanisms in brain development and disease. *Curr. Opin. Genet. Dev.* 65 42–46. 10.1016/j.gde.2020.05.006 32554106

[B6] Ayala-Mar S. Donoso-Quezada J. Gallo-Villanueva R. C. Perez-Gonzalez V. H. Gonzalez-Valdez J. (2019). Recent advances and challenges in the recovery and purification of cellular exosomes. *Electrophoresis* 40 3036–3049. 10.1002/elps.201800526 31373715 PMC6972601

[B7] Banks W. A. Sharma P. Bullock K. M. Hansen K. M. Ludwig N. Whiteside T. L. (2020). Transport of Extracellular Vesicles across the Blood-Brain Barrier: brain Pharmacokinetics and Effects of Inflammation. *Int. J. Mol. Sci.* 21:4407. 10.3390/ijms21124407 32575812 PMC7352415

[B8] Baroy T. Sorensen K. Lindeberg M. M. Frengen E. (2010). shRNA Expression Constructs Designed Directly from siRNA Oligonucleotide Sequences. *Mol. Biotechnol.* 45 116–120. 10.1007/s12033-010-9247-8 20119685

[B9] Bernard D. Prasanth K. V. Tripathi V. Colasse S. Nakamura T. Xuan Z. Y. (2010). A long nuclear-retained non-coding RNA regulates synaptogenesis by modulating gene expression. *EMBO J.* 29 3082–3093. 10.1038/emboj.2010.199 20729808 PMC2944070

[B10] Bloem B. R. Okun M. S. Klein C. (2021). Parkinson’s disease. *Lancet* 397 2284–2303. 10.1016/S0140-6736(21)00218-X 33848468

[B11] Bruce T. F. Slonecki T. J. Wang L. Huang S. S. Powell R. R. Marcus R. K. (2019). Exosome isolation and purification via hydrophobic interaction chromatography using a polyester, capillary-channeled polymer fiber phase. *Electrophoresis* 40 571–581. 10.1002/elps.201800417 30548636 PMC6881775

[B12] Cao X. Y. Lu J. M. Zhao Z. Q. Li M. C. Lu T. An X. S. (2017). MicroRNA biomarkers of Parkinson’s disease in serum exosome-like microvesicles. *Neurosci. Lett.* 644 94–99. 10.1016/j.neulet.2017.02.045 28223160

[B13] Charvin D. Medori R. Hauser R. A. Rascol O. (2018). Therapeutic strategies for Parkinson disease: beyond dopaminergic drugs. *Nat. Rev. Drug Discov.* 17 804–822. 10.1038/nrd.2018.184 30262889

[B14] Chen C. L. Zhang S. J. Wei Y. H. Sun X. B. (2021). LncRNA RMST Regulates Neuronal Apoptosis and Inflammatory Response via Sponging miR-150-5p in Parkinson’s Disease. *Neuroimmunomodulation* 29 55–62. 10.1159/000518212 34515176

[B15] Chen Q. H. Deng N. Lu K. Liao Q. Long X. Y. Gou D. M. (2021). Elevated plasma miR-133b and miR-221-3p as biomarkers for early Parkinson’s disease. *Sci. Rep.* 11:15268. 10.1038/s41598-021-94734-z 34315950 PMC8316346

[B16] Chen X. W. Lin B. C. Luo M. Z. Chu W. B. Li P. Liu H. L. (2021). Identifying circRNA- and lncRNA-associated-ceRNA networks in the hippocampi of rats exposed to PM2.5 using RNA-seq analysis. *Genomics* 113 193–204. 10.1016/j.ygeno.2020.12.025 33338629

[B17] Chen C. Y. Weng Y. H. Chien K. Y. Lin K. J. Yeh T. H. Cheng Y. P. (2012). (G2019S) LRRK2 activates MKK4-JNK pathway and causes degeneration of SN dopaminergic neurons in a transgenic mouse model of PD. *Cell Death Differ.* 19 1623–1633. 10.1038/cdd.2012.42 22539006 PMC3438494

[B18] Chen L. Yang J. X. Lu J. H. Cao S. S. Zhao Q. Yu Z. R. (2018). Identification of aberrant circulating miRNAs in Parkinson’s disease plasma samples. *Brain Behav.* 8:e00941. 10.1002/brb3.941 29670823 PMC5893342

[B19] Cheng J. Duan Y. Y. Zhang F. T. Shi J. Li H. Wang F. (2021). The Role of lncRNA TUG1 in the Parkinson Disease and Its Effect on Microglial Inflammatory Response. *Neuromol. Med.* 23 327–334. 10.1007/s12017-020-08626-y 33085068

[B20] Cheng X. Y. Xu S. Y. Zhang C. H. Qin K. Yan J. G. Shao X. Y. (2020). The BRCC3 regulated by Cdk5 promotes the activation of neuronal NLRP3 inflammasome in Parkinson’s disease models. *Biochem. Biophys. Res. Commun.* 522 647–654. 10.1016/j.bbrc.2019.11.141 31787240

[B21] Chistiakov D. A. Chistiakov A. A. (2017). alpha-Synuclein-carrying extracellular vesicles in Parkinson’s disease: deadly transmitters. *Acta Neurol. Belg.* 117 43–51. 10.1007/s13760-016-0679-1 27473175

[B22] Ciregia F. Urbani A. Palmisano G. (2017). Extracellular Vesicles in Brain Tumors and Neurodegenerative Diseases. *Front. Mol. Neurosci.* 10:276. 10.3389/fnmol.2017.00276 28912682 PMC5583211

[B23] Cokmus F. P. Ozmen E. Alkin T. Batir M. B. Cam F. S. (2019). Evaluation of serum MicroRNA expression profiles in patients with panic disorder. *Psychiatry Clin. Psychopharmacol.* 29 8–13. 10.1080/24750573.2018.1429844

[B24] Collier T. J. Redmond D. E. Steece-Collier K. Lipton J. W. Manfredsson F. P. (2016). Is Alpha-Synuclein Loss-of-Function a Contributor to Parkinsonian Pathology? Evidence from Non-human Primates. *Front. Neurosci.* 10:12. 10.3389/fnins.2016.00012 26858591 PMC4731516

[B25] Cooper J. M. Wiklander P. B. O. Nordin J. Z. Al-Shawi R. Wood M. J. Vithlani M. (2014). Systemic Exosomal siRNA Delivery Reduced Alpha-Synuclein Aggregates in Brains of Transgenic Mice. *Mov. Disord.* 29 1476–1485. 10.1002/mds.25978 25112864 PMC4204174

[B26] Cressatti M. Juwara L. Galindez J. M. Velly A. M. Nkurunziza E. S. Marier S. (2020). Salivary microR-153 and microR-223 Levels as Potential Diagnostic Biomarkers of Idiopathic Parkinson’s Disease. *Mov. Disord.* 35 468–477. 10.1002/mds.27935 31800144

[B27] Danzer K. M. Kranich L. R. Ruf W. P. Cagsal-Getkin O. Winslow A. R. Zhu L. Y. (2012). Exosomal cell-to-cell transmission of alpha synuclein oligomers. *Mol. Neurodegener.* 7:42. 10.1186/1750-1326-7-42 22920859 PMC3483256

[B28] Delpech J. C. Herron S. Botros M. B. Ikezu T. (2019). Neuroimmune Crosstalk through Extracellular Vesicles in Health and Disease. *Trends Neurosci.* 42 361–372. 10.1016/j.tins.2019.02.007 30926143 PMC6486849

[B29] Ding L. H. Yang X. N. Gao Z. B. Effah C. Y. Zhang X. J. Wu Y. J. (2021). A Holistic Review of the State-of-the-Art Microfluidics for Exosome Separation: an Overview of the Current Status, Existing Obstacles, and Future Outlook. *Small* 17:e2007174. 10.1002/smll.202007174 34047052

[B30] Dong X. Y. Zheng D. M. Nao J. F. (2020). Circulating Exosome microRNAs as Diagnostic Biomarkers of Dementia. *Front. Aging Neurosci.* 12:580199. 10.3389/fnagi.2020.580199 33093831 PMC7506134

[B31] Dos Santos M. C. T. Barreto-Sanz M. A. Correia B. R. S. Bell R. Widnall C. Perez L. T. (2018). miRNA-based signatures in cerebrospinal fluid as potential diagnostic tools for early stage Parkinson’s disease. *Oncotarget* 9 17455–17465. 10.18632/oncotarget.24736 29707120 PMC5915128

[B32] El-Agnaf O. M. A. Salem S. A. Paleologou K. E. Cooper L. J. Fullwood N. J. Gibson M. J. (2003). alpha-synuclein implicated in Parkinson’s disease is present in extracellular biological fluids, including human plasma. *FASEB J.* 17:1945. 10.1096/fj.03-0098fje 14519670

[B33] Elsharkasy O. M. Nordin J. Z. Hagey D. W. de Jong O. G. Schiffelers R. M. El Andaloussi S. (2020). Extracellular vesicles as drug delivery systems: why and how?. *Adv. Drug Deliv. Rev.* 159 332–343. 10.1016/j.addr.2020.04.004 32305351

[B34] Fang Y. W. Wan C. Wen Y. L. Wu Z. Pan J. Zhong M. (2021). Autism-associated synaptic vesicle transcripts are differentially expressed in maternal plasma exosomes of physiopathologic pregnancies. *J. Transl. Med.* 19:154. 10.1186/s12967-021-02821-6 33858444 PMC8051067

[B35] Fiedler J. Baker A. H. Dimmeler S. Heymans S. Mayr M. Thum T. (2018). Non-coding RNAs in vascular disease - from basic science to clinical applications: scientific update from the Working Group of Myocardial Function of the European Society of Cardiology. *Cardiovasc. Res.* 114 1281–1286. 10.1093/cvr/cvy121 29800267 PMC6054241

[B36] Figura M. Sitkiewicz E. Swiderska B. Milanowski L. Szlufik S. Koziorowski D. (2021). Proteomic Profile of Saliva in Parkinson’s Disease Patients: a Proof of Concept Study. *Brain Sci.* 11:661. 10.3390/brainsci11050661 34070185 PMC8158489

[B37] Finelli M. J. Paramo T. Pires E. Ryan B. J. Wade-Martins R. Biggin P. C. (2019). Oxidation Resistance 1 Modulates Glycolytic Pathways in the Cerebellum via an Interaction with Glucose-6-Phosphate Isomerase. *Mol. Neurobiol.* 56 1558–1577. 10.1007/s12035-018-1174-x 29905912 PMC6368252

[B38] Fraser K. B. Rawlins A. B. Clark R. G. Alcalay R. N. Standaert D. G. Liu N. J. (2016). Ser(P)-1292 LRRK2 in urinary exosomes is elevated in idiopathic Parkinson’s disease. *Mov. Disord.* 31 1543–1550. 10.1002/mds.26686 27297049 PMC5053851

[B39] Gao B. Y. Zhou S. T. Sun C. C. Cheng D. D. Zhang Y. Li X. T. (2020). Brain Endothelial Cell-Derived Exosomes Induce Neuroplasticity in Rats with Ischemia/Reperfusion Injury. *ACS Chem. Neurosci.* 11 2201–2213. 10.1021/acschemneuro.0c00089 32574032

[B40] Goetzl L. Darbinian N. Merabova N. (2019). Noninvasive assessment of fetal central nervous system insult: potential application to prenatal diagnosis. *Prenat. Diagn.* 39 609–615. 10.1002/pd.5474 31069822

[B41] Gomes A. R. Sangani N. B. Fernandes T. G. Diogo M. M. Curfs L. M. G. Reutelingsperger C. P. (2020). Extracellular Vesicles in CNS Developmental Disorders. *Int. J. Mol. Sci.* 21:9428. 10.3390/ijms21249428 33322331 PMC7763819

[B42] Gui Y. X. Liu H. Zhang L. S. Lv W. Hu X. Y. (2015). Altered microRNA profiles in cerebrospinal fluid exosome in Parkinson disease and Alzheimer disease. *Oncotarget* 6 37043–37053. 10.18632/oncotarget.6158 26497684 PMC4741914

[B43] Guo M. Wang J. Zhao Y. Feng Y. Han S. Dong Q. (2020). Microglial exosomes facilitate alpha-synuclein transmission in Parkinson’s disease. *Brain* 143 1476–1497. 10.1093/brain/awaa090 32355963 PMC7241957

[B44] Guo Y. D. Liu R. H. Zhou L. Y. Zhao H. Lv F. T. Liu L. B. (2020). Blood-brain-barrier penetrable thiolated paclitaxel-oligo (p-phenylene vinylene) nanomedicine with increased drug efficiency for glioblastoma treatment. *Nano Today* 35:100969. 10.1016/j.nantod.2020.100969

[B45] Gupta S. Rawat S. Arora V. Kottarath S. K. Dinda A. K. Vaishnav P. K. (2018). An improvised one-step sucrose cushion ultracentrifugation method for exosome isolation from culture supernatants of mesenchymal stem cells. *Stem Cell Res. Ther.* 9:180. 10.1186/s13287-018-0923-0 29973270 PMC6033286

[B46] Han C. Xiong N. Guo X. F. Huang J. S. Ma K. Liu L. (2019). Exosomes from patients with Parkinson’s disease are pathological in mice. *J. Mol. Med.* 97 1329–1344. 10.1007/s00109-019-01810-z 31302715

[B47] Hanan M. Simchovitz A. Yayon N. Vaknine S. Cohen-Fultheim R. Karmon M. (2020). A Parkinson’s disease CircRNAs Resource reveals a link between circSLC8A1 and oxidative stress. *EMBO Mol. Med.* 12:e11942. 10.15252/emmm.201911942 32715657 PMC7507321

[B48] Haney M. J. Klyachko N. L. Zhaoa Y. L. Gupta R. Plotnikova E. G. He Z. J. (2015). Exosomes as drug delivery vehicles for Parkinson’s disease therapy. *J. Control. Release* 207 18–30. 10.1016/j.jconrel.2015.03.033 25836593 PMC4430381

[B49] Harischandra D. S. Ghaisas S. Rokad D. Zamanian M. Jin H. Anantharam V. (2018). Environmental neurotoxicant manganese regulates exosome-mediated extracellular miRNAs in cell culture model of Parkinson’s disease: relevance to alpha-synuclein misfolding in metal neurotoxicity. *Neurotoxicology* 64 267–277. 10.1016/j.neuro.2017.04.007 28450057 PMC5654692

[B50] Harrell C. R. Volarevic A. Djonov V. Volarevic V. (2021). Mesenchymal Stem Cell-Derived Exosomes as New Remedy for the Treatment of Neurocognitive Disorders. *Int. J. Mol. Sci.* 22:1433. 10.3390/ijms22031433 33535376 PMC7867043

[B51] Hawley Z. C. E. Campos-Melo D. Droppelmann C. A. Strong M. J. (2017). MotomiRs: miRNAs in Motor Neuron Function and Disease. *Front. Mol. Neurosci.* 10:127. 10.3389/fnmol.2017.00127 28522960 PMC5415563

[B52] Ho D. H. Yi S. Seo H. Son I. Seol W. (2014). Increased DJ-1 in Urine Exosome of Korean Males with Parkinson’s Disease. *Biomed Res. Int.* 2014:704678. 10.1155/2014/704678 25478574 PMC4247948

[B53] Hoye M. L. Regan M. R. Jensen L. A. Lake A. M. Reddy L. V. Vidensky S. (2018). Motor neuron-derived microRNAs cause astrocyte dysfunction in amyotrophic lateral sclerosis. *Brain* 141 2561–2575. 10.1093/brain/awy182 30007309 PMC6113638

[B54] Iqbal S. Howard S. LoGrasso P. V. (2015). Serum- and Glucocorticoid-Inducible Kinase 1 Confers Protection in Cell-Based and in In Vivo Neurotoxin Models via the c-Jun N-Terminal Kinase Signaling Pathway. *Mol. Cell. Biol.* 35 1992–2006. 10.1128/Mcb.01510-14 25825522 PMC4420923

[B55] Izco M. Blesa J. Schleef M. Schmeer M. Porcari R. Al-Shawi R. (2019). Systemic Exosomal Delivery of shRNA Minicircles Prevents Parkinsonian Pathology. *Mol. Ther.* 27 2111–2122. 10.1016/j.ymthe.2019.08.010 31501034 PMC6904801

[B56] Izco M. Carlos E. Alvarez-Erviti L. (2021). The Two Faces of Exosomes in Parkinson’s Disease: from Pathology to Therapy. *Neuroscientist.* 10.1177/1073858421990001 [Epub ahead of print]. 33530851

[B57] Jalaludin I. Lubman D. M. Kim J. (2021). A guide to mass spectrometric analysis of extracellular vesicle proteins for biomarker discovery. *Mass Spectrom. Rev.* 10.1002/mas.21749 [Epub ahead of print]. 34747512

[B58] Janas A. M. Sapon K. Janas T. Stowell M. H. B. Janas T. (2016). Exosomes and other extracellular vesicles in neural cells and neurodegenerative diseases. *Biochim. Biophys. Acta Biomembr.* 1858 1139–1151. 10.1016/j.bbamem.2016.02.011 26874206

[B59] Jeppesen D. K. Fenix A. M. Franklin J. L. Higginbotham J. N. Zhang Q. Zimmerman L. J. (2019). Reassessment of Exosome Composition. *Cell* 177 428–445.e18. 10.1016/j.cell.2019.02.029 30951670 PMC6664447

[B60] Jiang Y. Liu J. Chen L. Z. Jin Y. Zhang G. P. Lin Z. H. (2019). Serum secreted miR-137-containing exosomes affects oxidative stress of neurons by regulating OXR1 in Parkinson’s disease. *Brain Res.* 1722:146331. 10.1016/j.brainres.2019.146331 31301273

[B61] Jucker M. Walker L. C. (2013). Self-propagation of pathogenic protein aggregates in neurodegenerative diseases. *Nature* 501 45–51. 10.1038/nature12481 24005412 PMC3963807

[B62] Jurado-Coronel J. C. Cabezas R. Rodriguez M. F. A. Echeverria V. Garcia-Segura L. M. Barreto G. E. (2018). Sex differences in Parkinson’s disease: features on clinical symptoms, treatment outcome, sexual hormones and genetics. *Front. Neuroendocrinol.* 50 18–30. 10.1016/j.yfrne.2017.09.002 28974386

[B63] Juzwik C. A. Drake S. Lecuyer M. A. Johnson R. M. Morquette B. Zhang Y. (2018). Neuronal microRNA regulation in Experimental Autoimmune Encephalomyelitis. *Sci. Rep.* 8:13437. 10.1038/s41598-018-31542-y 30194392 PMC6128870

[B64] Juzwik C. A. Drake S. S. Zhang Y. Paradis-Isler N. Sylvester A. Amar-Zifkin A. (2019). microRNA dysregulation in neurodegenerative diseases: a systematic review. *Prog. Neurobiol.* 182:101664. 10.1016/j.pneurobio.2019.101664 31356849

[B65] Kabaria S. Choi D. C. Chaudhuri A. D. Mouradian M. M. Junn E. (2015). Inhibition of miR-34b and miR-34c enhances alpha-synuclein expression in Parkinson’s disease. *FEBS Lett.* 589 319–325. 10.1016/j.febslet.2014.12.014 25541488 PMC4306645

[B66] Kalluri R. LeBleu V. S. (2020). The biology, function, and biomedical applications of exosomes. *Science* 367:eaau6977. 10.1126/science.aau6977 32029601 PMC7717626

[B67] Karampetsou M. Sykioti V. S. Leandrou E. Melachroinou K. Lambiris A. Giannelos A. (2020). Intrastriatal Administration of Exosome-Associated Pathological Alpha-Synuclein Is Not Sufficient by Itself to Cause Pathology Transmission. *Front. Neurosci.* 14:246. 10.3389/fnins.2020.00246 32372894 PMC7186405

[B68] Khoo S. K. Petillo D. Kang U. J. Resau J. H. Berryhill B. Linder J. (2012). Plasma-Based Circulating MicroRNA Biomarkers for Parkinson’s Disease. *J. Parkinsons Dis.* 2 321–331. 10.3233/Jpd-012144 23938262

[B69] Kim J. Inoue K. Ishii J. Vanti W. B. Voronov S. V. Murchison E. (2007). A microRNA feedback circuit in midbrain dopamine neurons. *Science* 317 1220–1224. 10.1126/science.1140481 17761882 PMC2782470

[B70] Kojima R. Bojar D. Rizzi G. Hamri G. C. E. El-Baba M. D. Saxena P. (2018). Designer exosomes produced by implanted cells intracerebrally deliver therapeutic cargo for Parkinson’s disease treatment. *Nat. Commun.* 9:1305. 10.1038/s41467-018-03733-8 29610454 PMC5880805

[B71] Kordower J. H. Chu Y. P. Hauser R. A. Freeman T. B. Olanow C. W. (2008). Lewy body-like pathology in long-term embryonic nigral transplants in Parkinson’s disease. *Nat. Med.* 14 504–506. 10.1038/nm1747 18391962

[B72] Kourembanas S. (2015). Exosomes: vehicles of intercellular signaling, biomarkers, and vectors of cell therapy. *Annu. Rev. Physiol.* 77 13–27. 10.1146/annurev-physiol-021014-071641 25293529

[B73] Kraus T. F. J. Haider M. Spanner J. Steinmaurer M. Dietinger V. Kretzschmar H. A. (2017). Altered Long Noncoding RNA Expression Precedes the Course of Parkinson’s Disease-a Preliminary Report. *Mol. Neurobiol.* 54 2869–2877. 10.1007/s12035-016-9854-x 27021022

[B74] Kuo M. C. Liu S. C. H. Hsu Y. F. Wu R. M. (2021). The role of noncoding RNAs in Parkinson’s disease: biomarkers and associations with pathogenic pathways. *J. Biomed. Sci.* 28:78. 10.1186/s12929-021-00775-x 34794432 PMC8603508

[B75] Lachenal G. Pernet-Gallay K. Chivet M. Hemming F. J. Belly A. Bodon G. (2011). Release of exosomes from differentiated neurons and its regulation by synaptic glutamatergic activity. *Mol. Cell. Neurosci.* 46 409–418. 10.1016/j.mcn.2010.11.004 21111824

[B76] Li Q. Wang Z. H. Xing H. Wang Y. Guo Y. (2021). Exosomes derived from miR-188-3p-modified adipose-derived mesenchymal stem cells protect Parkinson’s disease. *Mol. Ther. Nucleic Acids* 23 1334–1344. 10.1016/j.omtn.2021.01.022 33717653 PMC7920810

[B77] Li S. S. Yao J. P. Xie M. J. Liu Y. N. Zheng M. (2018). Exosomal miRNAs in hepatocellular carcinoma development and clinical responses. *J. Hematol. Oncol.* 11:54. 10.1186/s13045-018-0579-3 29642941 PMC5896112

[B78] Li X. Q. Zhao C. Z. (2021). Catalpol Exerts Neuroprotective Effect on 1-Methyl-4-Phenyl-1,2,3,6-Tetrahydropyridine (MPTP)-Induced Mouse Model of Parkinson’s Disease and the Underlying Mechanisms. *J. Biomater. Tissue Eng.* 11 1506–1516. 10.1166/jbt.2021.2713

[B79] Li Y. Li W. Liu C. Yan M. Raman I. Du Y. (2014). Delivering Oxidation Resistance-1 (OXR1) to Mouse Kidney by Genetic Modified Mesenchymal Stem Cells Exhibited Enhanced Protection against Nephrotoxic Serum Induced Renal Injury and Lupus Nephritis. *J. Stem Cell Res. Ther.* 4:231. 10.4172/2157-7633.1000231 25995969 PMC4435960

[B80] Liang H. Chen J. (2020). Evolution of blood–brain barrier in brain diseases and related systemic nanoscale brain-targeting drug delivery strategies. *Acta Pharm. Sin. B*. 11 2306–2325. 10.1016/j.apsb.2020.11.023 34522589 PMC8424230

[B81] Liu H. Y. Huang C. M. Hung Y. F. Hsueh Y. P. (2015). The microRNAs Let7c and miR21 are recognized by neuronal Toll-like receptor 7 to restrict dendritic growth of neurons. *Exp. Neurol.* 269 202–212. 10.1016/j.expneurol.2015.04.011 25917529

[B82] Liu L. Y. Li Y. Peng H. Liu R. Y. Ji W. H. Shi Z. Y. (2020). Targeted exosome coating gene-chem nanocomplex as “nanoscavenger” for clearing alpha-synuclein and immune activation of Parkinson’s disease. *Sci. Adv.* 6:eaba3967. 10.1126/sciadv.aba3967 33310840 PMC7732192

[B83] Lopez Gonzalez I. Garcia-Esparcia P. Llorens F. Ferrer I. (2016). Genetic and Transcriptomic Profiles of Inflammation in Neurodegenerative Diseases: Alzheimer, Parkinson, Creutzfeldt-Jakob and Tauopathies. *Int. J. Mol. Sci.* 17:206. 10.3390/ijms17020206 26861289 PMC4783939

[B84] Lun P. Ji T. Wan D.-H. Liu X. Chen X.-D. Yu S. (2022). HOTTIP downregulation reduces neuronal damage and microglial activation in Parkinson’s disease cell and mouse models. *Neural Regen. Res.* 17 887–897. 10.4103/1673-5374.322475 34472490 PMC8530116

[B85] Ma N. N. Zhang W. Wan J. (2020). Research Progress on circRNA in Nervous System Diseases. *Curr. Alzheimer Res.* 17 687–697. 10.2174/1567205017666201111114928 33176648

[B86] Ma X. L. Zhang H. Yin H. L. Geng S. Liu Y. J. Liu C. (2021). Up-regulated microRNA-218-5p ameliorates the damage of dopaminergic neurons in rats with Parkinson’s disease via suppression of LASP1. *Brain Res. Bull.* 166 92–101. 10.1016/j.brainresbull.2020.10.019 33144090

[B87] Margis R. Margis R. Rieder C. R. M. (2011). Identification of blood microRNAs associated to Parkinsonis disease. *J. Biotechnol.* 152 96–101. 10.1016/j.jbiotec.2011.01.023 21295623

[B88] Marrugo-Ramirez J. Rodriguez-Nunez M. Marco M. P. Mir M. Samitier J. (2021). Kynurenic Acid Electrochemical Immunosensor: blood-Based Diagnosis of Alzheimer’s Disease. *Biosensors* 11:20. 10.3390/bios11010020 33445512 PMC7827041

[B89] McMillan K. J. Murray T. K. Bengoa-Vergniory N. Cordero-Llana O. Cooper J. Buckley A. (2017). Loss of MicroRNA-7 Regulation Leads to alpha-Synuclein Accumulation and Dopaminergic Neuronal Loss In Vivo. *Mol. Ther.* 25 2404–2414. 10.1016/j.ymthe.2017.08.017 28927576 PMC5628933

[B90] Men Y. Q. Yelick J. Jin S. J. Tian Y. Chiang M. S. R. Higashimori H. (2019). Exosome reporter mice reveal the involvement of exosomes in mediating neuron to astroglia communication in the CNS. *Nat. Commun.* 10:4136. 10.1038/s41467-019-11534-w 31515491 PMC6742670

[B91] Meng Y. Ding J. Li C. Fan H. He Y. Qiu P. (2020). Transfer of pathological alpha-synuclein from neurons to astrocytes via exosomes causes inflammatory responses after METH exposure. *Toxicol. Lett.* 331 188–199. 10.1016/j.toxlet.2020.06.016 32569805

[B92] Morel L. Regan M. Higashimori H. Ng S. K. Esau C. Vidensky S. (2013). Neuronal Exosomal miRNA-dependent Translational Regulation of Astroglial Glutamate Transporter GLT1. *J. Biol. Chem.* 288 7105–7116. 10.1074/jbc.M112.410944 23364798 PMC3591620

[B93] Nasirishargh A. Kumar P. Ramasubramanian L. Clark K. Hao D. K. Lazar S. V. (2021). Exosomal microRNAs from mesenchymal stem/stromal cells: biology and applications in neuroprotection. *World J. Stem Cells* 13 776–794. 10.4252/wjsc.v13.i7.776 34367477 PMC8316862

[B94] Nie C. Sun Y. Z. Zhen H. F. Guo M. Ye J. Y. Liu Z. L. (2020). Differential Expression of Plasma Exo-miRNA in Neurodegenerative Diseases by Next-Generation Sequencing. *Front. Neurosci.* 14:438. 10.3389/fnins.2020.00438 32457573 PMC7227778

[B95] Osmanovic A. Ranxha G. Kumpe M. Muschen L. Binz C. Wiehler F. (2020). Treatment expectations and patient-reported outcomes of nusinersen therapy in adult spinal muscular atrophy. *J. Neurol.* 267 2398–2407. 10.1007/s00415-020-09847-8 32361837 PMC7359174

[B96] Ozdilek B. Demircan B. (2020). Serum microRNA expression levels in Turkish patients with Parkinson’s disease. *Int. J. Neurosci.* 131 1181–1189. 10.1080/00207454.2020.1784165 32546033

[B97] Pan T. H. Kondo S. Le W. D. Jankovic J. (2008). The role of autophagy-lysosome pathway in neurodegeneration associated with Parkinson’s disease. *Brain* 131 1969–1978. 10.1093/brain/awm318 18187492

[B98] Parsi S. Smith P. Y. Goupil C. Dorval V. Hebert S. S. (2015). Preclinical Evaluation of miR-15/107 Family Members as Multifactorial Drug Targets for Alzheimer’s Disease. *Mol. Ther. Nucleic Acids* 4:e256. 10.1038/mtna.2015.33 26440600 PMC4881761

[B99] Pashova A. Work L. M. Nicklin S. A. (2020). The role of extracellular vesicles in neointima formation post vascular injury. *Cell. Signal.* 76:109783. 10.1016/j.cellsig.2020.109783 32956789

[B100] Patel N. A. Moss L. D. Lee J. Y. Tajiri N. Acosta S. Hudson C. (2018). Long noncoding RNA MALAT1 in exosomes drives regenerative function and modulates inflammation-linked networks following traumatic brain injury. *J. Neuroinflammation* 15:204. 10.1186/s12974-018-1240-3 30001722 PMC6044101

[B101] Peng H. Ji W. H. Zhao R. C. Yang J. Lu Z. G. Li Y. (2020). Exosome: a significant nano-scale drug delivery carrier. *J. Mater. Chem. B* 8 7591–7608. 10.1039/d0tb01499k 32697267

[B102] Popovic M. de Marco A. (2018). Canonical and selective approaches in exosome purification and their implications for diagnostic accuracy. *Transl. Cancer Res.* 7 S209–S225. 10.21037/tcr.2017.08.44

[B103] Prieto-Fernandez E. Aransay A. M. Royo F. Gonzalez E. Lozano J. J. Santos-Zorrozua B. (2019). A Comprehensive Study of Vesicular and Non-Vesicular miRNAs from a Volume of Cerebrospinal Fluid Compatible with Clinical Practice. *Theranostics* 9 4567–4579. 10.7150/thno.31502 31367240 PMC6643433

[B104] Qiu L. F. Zhang W. Tan E. K. Zeng L. (2014). Deciphering the Function and Regulation of microRNAs in Alzheimer’s Disease and Parkinson’s Disease. *ACS Chem. Neurosci.* 5 884–894. 10.1021/cn500149w 25210999

[B105] Qiu Y. Li P. Y. Zhang Z. P. Wu M. H. (2021). Insights Into Exosomal Non-Coding RNAs Sorting Mechanism and Clinical Application. *Front. Oncol.* 11:664904. 10.3389/fonc.2021.664904 33987099 PMC8111219

[B106] Qu M. K. Lin Q. Huang L. Y. Fu Y. Wang L. Y. He S. S. (2018). Dopamine-loaded blood exosomes targeted to brain for better treatment of Parkinson’s disease. *J. Control. Release* 287 156–166. 10.1016/j.jconrel.2018.08.035 30165139

[B107] Quan Y. Wang J. Wang S. Zhao J. Z. (2020). Association of the Plasma Long Non-coding RNA MEG3 With Parkinson’s Disease. *Front. Neurol.* 11:532891. 10.3389/fneur.2020.532891 33329296 PMC7732627

[B108] Reddy A. P. Ravichandran J. Carkaci-Salli N. (2020). Neural regeneration therapies for Alzheimer’s and Parkinson’s disease-related disorders. *Biochim. Biophys. Acta Mol. Basis Dis.* 1866:165506. 10.1016/j.bbadis.2019.06.020 31276770

[B109] Reichenstein I. Eitan C. Diaz-Garcia S. Haim G. Magen I. Siany A. (2019). Human genetics and neuropathology suggest a link between miR-218 and amyotrophic lateral sclerosis pathophysiology. *Sci. Transl. Med.* 11:eaav5264. 10.1126/scitranslmed.aav5264 31852800 PMC7057809

[B110] Rennert O. M. Ziats M. N. (2017). Editorial: non-Coding RNAs in Neurodevelopmental Disorders. *Front. Neurol.* 8:629. 10.3389/fneur.2017.00629 29230193 PMC5712063

[B111] Roser A. E. Gomes L. C. Schunemann J. Maass F. Lingor P. (2018). Circulating miRNAs as Diagnostic Biomarkers for Parkinson’s Disease. *Front. Neurosci.* 12:625. 10.3389/fnins.2018.00625 30233304 PMC6135037

[B112] Schaeffer E. Kluge A. Bottner M. Zunke F. Cossais F. Berg D. (2020). Alpha Synuclein Connects the Gut-Brain Axis in Parkinson’s Disease Patients - A View on Clinical Aspects, Cellular Pathology and Analytical Methodology. *Front. Cell Dev. Biol.* 8:573696. 10.3389/fcell.2020.573696 33015066 PMC7509446

[B113] Scoles D. R. Minikel E. V. Pulst S. M. (2019). Antisense oligonucleotides. *Neurol. Genet.* 5:e323. 10.1212/NXG.0000000000000323 31119194 PMC6501637

[B114] Scoles D. R. Pulst S. M. (2019). Antisense therapies for movement disorders. *Mov. Disord.* 34 1112–1119. 10.1002/mds.27782 31283857

[B115] Shakespear N. Ogura M. Yamaki J. Homma Y. (2020). Astrocyte-Derived Exosomal microRNA miR-200a-3p Prevents MPP+-Induced Apoptotic Cell Death Through Down-Regulation of MKK4. *Neurochem. Res.* 45 1020–1033. 10.1007/s11064-020-02977-5 32016794

[B116] Sharma A. Jha N. K. Dahiya K. Singh V. K. Chaurasiya K. Jha A. N. (2020). Nanoparticulate RNA delivery systems in cancer. *Cancer Rep.* 3:e1271. 10.1002/cnr2.1271 32729987 PMC7941463

[B117] Sheng P. K. Flood K. A. Xie M. Y. (2020). Short Hairpin RNAs for Strand-Specific Small Interfering RNA Production. *Front. Bioeng. Biotechnol.* 8:940. 10.3389/fbioe.2020.00940 32850763 PMC7427337

[B118] Shi C. H. Zhang L. Qin C. (2017). Long non-coding RNAs in brain development, synaptic biology, and Alzheimer’s disease. *Brain Res. Bull.* 132 160–169. 10.1016/j.brainresbull.2017.03.010 28347717

[B119] Shi M. Liu C. Cook T. J. Bullock K. M. Zhao Y. Ginghina C. (2014). Plasma exosomal alpha-synuclein is likely CNS-derived and increased in Parkinson’s disease. *Acta Neuropathol.* 128 639–650. 10.1007/s00401-014-1314-y 24997849 PMC4201967

[B120] Shim K. H. Go H. G. Bae H. Jeong D. E. Kim D. Youn Y. C. (2021). Decreased Exosomal Acetylcholinesterase Activity in the Plasma of Patients With Parkinson’s Disease (vol 13, 665400, 2021). *Front. Aging Neurosci.* 13:665400. 10.3389/fnagi.2021.726525 34122043 PMC8193230

[B121] Shin W. H. Chung K. C. (2020). Death-associated Protein Kinase 1 Phosphorylates alpha-Synuclein at Ser129 and Exacerbates Rotenone-induced Toxic Aggregation of alpha-Synuclein in Dopaminergic SH-SY5Y Cells. *Exp. Neurobiol.* 29 207–218. 10.5607/en20014 32624505 PMC7344377

[B122] Soreq L. Guffanti A. Salomonis N. Simchovitz A. Israel Z. Bergman H. (2014). Long Non-Coding RNA and Alternative Splicing Modulations in Parkinson’s Leukocytes Identified by RNA Sequencing. *PLoS Comput. Biol.* 10:e1003517. 10.1371/journal.pcbi.1003517 24651478 PMC3961179

[B123] Stanga S. Boido M. Kienlen-Campard P. (2021). How to Build and to Protect the Neuromuscular Junction: the Role of the Glial Cell Line-Derived Neurotrophic Factor. *Int. J. Mol. Sci.* 22:136. 10.3390/ijms22010136 33374485 PMC7794999

[B124] Su Z. D. Huang Y. Zhang Z. Y. Zhao Y. W. Wang D. Chen W. (2018). iLoc-lncRNA: predict the subcellular location of lncRNAs by incorporating octamer composition into general PseKNC. *Bioinformatics* 34 4196–4204. 10.1093/bioinformatics/bty508 29931187

[B125] Sulaiman S. A. Muhsin N. I. A. Arshad A. R. Nazarie W. F. W. M. Jamal R. Ibrahim N. M. (2020). Differential expression of circulating miRNAs in Parkinson’s disease patients: potential early biomarker?. *Neurol. Asia* 25 319–329.

[B126] Sun F. Y. Deng Y. L. Han X. W. Liu Q. Q. Zhang P. Manzoor R. (2019). A secret that underlies Parkinson’s disease: the damaging cycle. *Neurochem. Int.* 129:104484. 10.1016/j.neuint.2019.104484 31173779

[B127] Sweeney M. D. Sagare A. P. Zlokovic B. V. (2018). Blood-brain barrier breakdown in Alzheimer disease and other neurodegenerative disorders. *Nat. Rev. Neurol.* 14 133–150. 10.1038/nrneurol.2017.188 29377008 PMC5829048

[B128] Taghizadeh E. Gheibihayat S. M. Taheri F. Afshani S. M. Farahani N. Saberi A. (2021). LncRNAs as putative biomarkers and therapeutic targets for Parkinson’s disease. *Neurol. Sci.* 42 4007–4015. 10.1007/s10072-021-05408-7 34254198

[B129] Tan C. L. Plotkin J. L. Veno M. T. von Schimmelmann M. Feinberg P. Mann S. (2013). MicroRNA-128 Governs Neuronal Excitability and Motor Behavior in Mice. *Science* 342 1254–1258. 10.1126/science.1244193 24311694 PMC3932786

[B130] Thery C. Witwer K. W. Aikawa E. Alcaraz M. J. Anderson J. D. Andriantsitohaina R. (2018). Minimal information for studies of extracellular vesicles 2018 (MISEV2018): a position statement of the International Society for Extracellular Vesicles and update of the MISEV2014 guidelines. *J. Extracell. Vesicles* 7:1535750. 10.1080/20013078.2018.1535750 30637094 PMC6322352

[B131] Valadi H. Ekstrom K. Bossios A. Sjostrand M. Lee J. J. Lotvall J. O. (2007). Exosome-mediated transfer of mRNAs and microRNAs is a novel mechanism of genetic exchange between cells. *Nat. Cell Biol.* 9 654–659. 10.1038/ncb1596 17486113

[B132] Vargas-Medrano J. Yang B. Garza N. T. Segura-Ulate I. Perez R. G. (2019). Up-regulation of protective neuronal MicroRNAs by FTY720 and novel FTY720-derivatives. *Neurosci. Lett.* 690 178–180. 10.1016/j.neulet.2018.10.040 30359694 PMC7952001

[B133] Vijiaratnam N. Simuni T. Bandmann O. Morris H. R. Foltynie T. (2021). Progress towards therapies for disease modification in Parkinson’s disease. *Lancet Neurol.* 20 559–572. 10.1016/S1474-4422(21)00061-2 34146514

[B134] Vilaca-Faria H. Salgado A. J. Teixeira F. G. (2019). Mesenchymal Stem Cells-derived Exosomes: a New Possible Therapeutic Strategy for Parkinson’s Disease?. *Cells* 8:118. 10.3390/cells8020118 30717429 PMC6406999

[B135] Volkert M. R. Crowley D. J. (2020). Preventing Neurodegeneration by Controlling Oxidative Stress: the Role of OXR1. *Front. Neurosci.* 14:611904. 10.3389/fnins.2020.611904 33384581 PMC7770112

[B136] Volkert M. R. Elliott N. A. Housman D. E. (2000). Functional genomics reveals a family of eukaryotic oxidation protection genes. *Proc. Natl. Acad. Sci. U. S. A.* 97 14530–14535. 10.1073/pnas.260495897 11114193 PMC18953

[B137] Wang Q. Han C. L. Wang K. L. Sui Y. P. Li Z. B. Chen N. (2020). Integrated analysis of exosomal lncRNA and mRNA expression profiles reveals the involvement of lnc-MKRN2-42:1 in the pathogenesis of Parkinson’s disease. *CNS Neurosci. Ther.* 26 527–537. 10.1111/cns.13277 31814304 PMC7163584

[B138] Wen Z. Shu Y. Gao C. Y. Wang X. M. Qi G. J. Zhang P. (2014). CDK5-mediated phosphorylation and autophagy of RKIP regulate neuronal death in Parkinson’s disease. *Neurobiol. Aging* 35 2870–2880. 10.1016/j.neurobiolaging.2014.05.034 25104559

[B139] Willis C. L. Camire R. B. Brule S. A. Ray D. E. (2013). Partial Recovery of the Damaged Rat Blood-Brain Barrier Is Mediated by Adherens Junction Complexes, Extracellular Matrix Remodeling and Macrophage Infiltration Following Focal Astrocyte Loss. *Neuroscience* 250 773–785. 10.1016/j.neuroscience.2013.06.061 23845748 PMC4002262

[B140] Wong A. S. L. Lee R. H. K. Cheung A. Y. Yeung P. K. Chung S. K. Cheung Z. H. (2011). Cdk5-mediated phosphorylation of endophilin B1 is required for induced autophagy in models of Parkinson’s disease. *Nat. Cell Biol.* 13 568–79. 10.1038/ncb2217 21499257

[B141] Xia Y. Zhang G. X. Han C. Ma K. Guo X. F. Wan F. (2019). Microglia as modulators of exosomal alpha-synuclein transmission. *Cell Death Dis.* 10:174. 10.1038/s41419-019-1404-9 30787269 PMC6382842

[B142] Yan Y. Q. Fang Y. Zheng R. Pu J. L. Zhang B. R. (2020). NLRP3 Inflammasomes in Parkinson’s disease and their Regulation by Parkin. *Neuroscience* 446 323–334. 10.1016/j.neuroscience.2020.08.004 32795556

[B143] Yang J. Luo S. Zhang J. Yu T. Fu Z. Zheng Y. (2021). Exosome-mediated delivery of antisense oligonucleotides targeting alpha-synuclein ameliorates the pathology in a mouse model of Parkinson’s disease. *Neurobiol. Dis.* 148:105218. 10.1016/j.nbd.2020.105218 33296726

[B144] Yang X. Q. Wu X. Yang Y. Gu T. Hong L. J. Zheng E. Q. (2019). Improvement of developmental competence of cloned male pig embryos by short hairpin ribonucleic acid (shRNA) vector-based but not small interfering RNA (siRNA)-mediated RNA interference (RNAi) of Xist expression. *J. Reprod. Dev.* 65 533–539. 10.1262/jrd.2019-070 31631092 PMC6923154

[B145] Yao Y. F. Qu M. W. Li G. C. Zhang F. B. Rui H. C. (2018). Circulating exosomal miRNAs as diagnostic biomarkers in Parkinson’s disease. *Eur. Rev. Med. Pharmacol. Sci.* 22 5278–5283. 10.26355/eurrev_201808_15727 30178852

[B146] Yelick J. Men Y. Q. Jin S. J. Seo S. Espejo-Porras F. Yang Y. J. (2020). Elevated exosomal secretion of miR-124-3p from spinal neurons positively associates with disease severity in ALS. *Exp. Neurol.* 333:113414. 10.1016/j.expneurol.2020.113414 32712030 PMC7502520

[B147] Yin J. X. Zeng A. L. Zhang Z. R. Shi Z. M. Yan W. You Y. P. (2019). Exosomal transfer of miR-1238 contributes to temozolomide-resistance in glioblastoma. *EBioMedicine* 42 238–251. 10.1016/j.ebiom.2019.03.016 30917935 PMC6491393

[B148] Yu H. Y. Sun T. An J. Wen L. L. Liu F. Bu Z. Q. (2020). Potential Roles of Exosomes in Parkinson’s Disease: from Pathogenesis, Diagnosis, and Treatment to Prognosis. *Front. Cell Dev. Biol.* 8:86. 10.3389/fcell.2020.00086 32154247 PMC7047039

[B149] Zhang G. P. Chen L. Z. Liu J. Jin Y. Lin Z. H. Du S. (2020). HIF-1 alpha/microrNA-128-3p axis protects hippocampal neurons from apoptosis via the Axin1-meaiated Wnt/beta-catenin signaling pathway in Parkinson’s disease moaeis. *Aging* 12 4067–4081. 10.18632/aging.102636 32167488 PMC7093183

[B150] Zhang S. F. Gao J. Liu C. M. (2019). The Role of Non-Coding RNAs in Neurodevelopmental Disorders. *Front. Genet.* 10:1033. 10.3389/fgene.2019.01033 31824553 PMC6882276

[B151] Zhang Z. Cheng Y. (2014). miR-16-1 Promotes the Aberrant alpha-Synuclein Accumulation in Parkinson Disease via Targeting Heat Shock Protein 70. *Sci. World J*. 2014:938348. 10.1155/2014/938348 25054189 PMC4094852

[B152] Zhao A. N. Li Y. Y. Niu M. Y. Li G. L. Luo N. D. Zhou L. C. (2020). SNCA Hypomethylation in Rapid Eye Movement Sleep Behavior Disorder Is a Potential Biomarker for Parkinson’s Disease. *J. Parkinsons Dis.* 10 1023–1031. 10.3233/Jpd-201912 32444558

[B153] Zhao Z. H. Chen Z. T. Zhou R. L. Zhang X. Ye Q. Y. Wang Y. Z. (2019). Increased DJ-1 and alpha-Synuclein in Plasma Neural-Derived Exosomes as Potential Markers for Parkinson’s Disease. *Front. Aging Neurosci.* 10:438. 10.3389/fnagi.2018.00438 30692923 PMC6339871

[B154] Zheng T. T. Zhang Z. X. (2021). Activated microglia facilitate the transmission of alpha-synuclein in Parkinson’s disease. *Neurochem. Int.* 148:105094. 10.1016/j.neuint.2021.105094 34097990

[B155] Zhou L. Yang L. Li Y. J. Mei R. Yu H. L. Gong Y. (2018). MicroRNA-128 Protects Dopamine Neurons from Apoptosis and Upregulates the Expression of Excitatory Amino Acid Transporter 4 in Parkinson’s Disease by Binding to AXIN1. *Cell. Physiol. Biochem.* 51 2275–2289. 10.1159/000495872 30537735

[B156] Zhou Y. Zhao W. J. Quan W. Qiao C. M. Cui C. Hong H. (2021). Dynamic changes of activated AHR in microglia and astrocytes in the substantia nigra-striatum system in an MPTP-induced Parkinson’s disease mouse model. *Brain Res. Bull.* 176 174–183. 10.1016/j.brainresbull.2021.08.013 34478811

[B157] Zou J. Guo Y. Wei L. Yu F. Yu B. Xu A. D. (2020). Long Noncoding RNA POU3F3 and alpha-Synuclein in Plasma L1CAM Exosomes Combined with beta-Glucocerebrosidase Activity: potential Predictors of Parkinson’s Disease. *Neurotherapeutics* 17 1104–1119. 10.1007/s13311-020-00842-5 32236821 PMC7609611

